# The Regulator OmpR in *Yersinia enterocolitica* Participates in Iron Homeostasis by Modulating Fur Level and Affecting the Expression of Genes Involved in Iron Uptake

**DOI:** 10.3390/ijms22031475

**Published:** 2021-02-02

**Authors:** Karolina Jaworska, Marta Ludwiczak, Emilia Murawska, Adrianna Raczkowska, Katarzyna Brzostek

**Affiliations:** Department of Molecular Microbiology, Institute of Microbiology, Faculty of Biology, University of Warsaw, Miecznikowa 1, 02-096 Warsaw, Poland; kjaworska@biol.uw.edu.pl (K.J.); m.ludwiczak@lmu.de (M.L.); emurawska@biol.uw.edu.pl (E.M.); kbrzostek@biol.uw.edu.pl (K.B.)

**Keywords:** *Yersinia enterocolitica*, OmpR regulator, Fur repressor, iron homeostasis, *fecA*, *fepA*, *feoA*

## Abstract

In this study, we found that the loss of OmpR, the response regulator of the two-component EnvZ/OmpR system, increases the cellular level of Fur, the master regulator of iron homeostasis in *Y. enterocolitica*. Furthermore, we demonstrated that transcription of the *fur* gene from the YeP*fur* promoter is subject to negative OmpR-dependent regulation. Four putative OmpR-binding sites (OBSs) were indicated by in silico analysis of the *fur* promoter region, and their removal affected OmpR-dependent *fur* expression. Moreover, OmpR binds specifically to the predicted OBSs which exhibit a distinct hierarchy of binding affinity. Finally, the data demonstrate that OmpR, by direct binding to the promoters of the *fecA*, *fepA* and *feoA* genes, involved in the iron transport and being under Fur repressor activity, modulates their expression. It seems that the negative effect of OmpR on *fecA* and *fepA* transcription is sufficient to counteract the indirect, positive effect of OmpR resulting from decreasing the Fur repressor level. The expression of *feoA* was positively regulated by OmpR and this mode of action seems to be direct and indirect. Together, the expression of *fecA*, *fepA* and *feoA* in *Y. enterocolitica* has been proposed to be under a complex mode of regulation involving OmpR and Fur regulators.

## 1. Introduction

*Yersinia enterocolitica* is a Gram-negative bacterium that exhibits a dual lifestyle, existing as both a pathogen of a broad range of animals and a saprophyte widespread in nature [1,2]. In humans, it causes yersiniosis, an important zoonotic gastrointestinal disease that occurs worldwide [3]. *Y. enterocolitica* is a heterogeneous species comprising six biotypes (1A, 1B, 2, 3, 4 and 5) that vary in pathogenicity due to the synthesis of different virulence factors [4,5]. The survival and growth of pathogenic bacteria outside and inside the host organism depends on iron availability [6]. However, at physiological pH in an aerobic environment, ferric ions (Fe^3+^) are essentially insoluble and unavailable for use by bacterial cells. Conversely, at low pH and in anaerobic environments, iron can switch from the insoluble ferric form to the more soluble ferrous (Fe^2+^) form. *Y. enterocolitica* and other pathogens encounter an additional obstacle in their quest for inorganic iron in the form of iron-binding compounds in the host organism, which control the availability of this essential trace nutrient [7]. To obtain the quantities of iron necessary for growth, pathogenic *Y. enterocolitica* have developed diverse and complex acquisition systems to acquire iron from different sources [8,9,10,11,12,13,14,15]. To obtain ferric iron, highly pathogenic strains of *Y. enterocolitica* (biotype 1B) synthesize the siderophore yersiniabactin, a high-affinity Fe^3+^-specific chelator [16]. Less pathogenic strains are unable to synthesize yersiniabactin and therefore import foreign siderophores complexed with Fe^3+^ via TonB-dependent OM transporters/receptors such as FecA, FepA, FoxA and FcuA [10,17]. Another mechanism operating in *Y. enterocolitica* is a system for acquiring iron from heme or hemoproteins, which employs the OM receptor HemR [8,15]. The uptake of ferrous iron can be achieved independently of siderophores and Gram-negative bacteria are equipped with an Fe^2+^ active transport system located in the cytoplasmic membrane (Feo system) [18]. Ferrous iron is thought to diffuse freely through the OM porins into the periplasm from where it is transported into the cytoplasm via the FeoABC system [19]. Systems homologous to FeoABC have been identified in pathogenic *Yersinia* species [20].

The concentration of iron in the environment controls the growth and cellular metabolism of bacteria [21]. However, while an iron shortage restricts bacterial growth, excessive intracellular accumulation of iron can be deleterious, damaging bacteria through the generation of reactive oxygen species by the Fenton reaction [22]. The precise control of iron acquisition, storage and utilization that ensures bacterial survival in a changing environment is coordinated by the Ferric-uptake regulator (Fur). Fur is an Fe^2+^-responsive transcription factor that controls the expression of genes involved in diverse cellular mechanisms associated with iron homeostasis. The Fur-dependent regulation of gene expression has been best studied in *Escherichia coli* K-12 [23,24]. Under iron-replete conditions, Fur binds ferrous iron (Fe^2+^), leading to its activation and binding of a 19-bp consensus DNA sequence (Fur box), located in the promoters of iron-regulated genes/operons required for iron acquisition or transport, which represses their transcription [25,26]. Under iron starvation conditions, the repression of Fur-regulated genes is relieved by dissociation of the ferrous ion from the Fur protein [27].

Since the original description of Fur in *E. coli* K-12 [28,29], this regulator has been recognized in many other bacterial species including human pathogens. The functions of Fur extend far beyond the regulation of iron homeostasis and include important roles in the defense against oxidative stress [30,31], carbon metabolism [9,32] and the acid tolerance response [33]. Moreover, Fur seems to be involved in other functions that are unrelated to the regulation of transcription and occur irrespective of the iron status [34]. Genome-wide computational analyses have revealed a number of potentially Fur-controlled genes, which indicates that Fur has an extremely broad range of influence [24]. Thus, the full picture of Fur activity is much more complex than was previously thought [35,36,37,38].

Based on experimental and bioinformatic analyses, Fur homologs have been identified in pathogenic *Yersinia* species, i.e., the plague bacillus *Y. pestis* and enteropathogen *Y. pseudotuberculosis* [14,39,40]. Almost all of the iron scavenging mechanisms in *Y. pestis* were shown to be controlled by Fur [41]. In addition, the identification of a Fur regulon in *Y. pestis* affirmed its role as a global regulator of gene expression [41,42]. In *Y. enterocolitica*, all genes required for the synthesis and transport of yersiniabactin, encoded within a high-pathogenicity island (HPI) found in *Y. enterocolitica* biotype 1B and responsible for its increased pathogenicity, are repressed by Fur under iron-replete conditions [14,43]. Other *Y. enterocolitica* genes subject to Fur-dependent regulation in response to iron concentrations encode outer membrane receptors and transport systems for the foreign siderophores ferrichrome, ferrioxamine B and E and enterobactin [10,17].

In Gram-negative bacteria, active Fe^2+^-Fur protein levels are regulated by *fur* gene expression and the intracellular labile Fe^2+^ pool [44]. The expression of *fur* is subject to complex regulation in response to environmental conditions such as the iron concentration, the available sources of carbon, oxidative stress and pH [45,46,47,48]. It has been shown that *fur* transcription in *E. coli* is autoregulated, and iron starvation slightly increases *fur* expression. Moderate autoregulation by the Fur protein occurs in most Enterobacteriaceae [46,49,50]. In *E. coli*, OxyR and SoxRS, two oxidative stress response regulators, activate *fur* expression [47]. Another important layer of *fur* regulation in *E. coli* is post-transcriptional downregulation involving the non-coding sRNA RyhB [51].

Two-component signal transduction systems (TCSs) are widespread in prokaryotes and play vital roles in adaptation to environmental changes by modulating bacterial gene expression [52,53]. The EnvZ/OmpR system of *E. coli*, an archetype of TCSs, comprises the EnvZ transmembrane sensor kinase and the transcriptional response regulator OmpR. As a classical response regulator, OmpR has an N-terminal receiver domain with a conserved Asp residue at position 55 and a C-terminal helix-turn-helix domain for DNA binding. It can regulate transcription in a positive or negative way by binding to consensus sequence elements that occur at different locations in promoter regions [54,55,56]. It has been shown that the EnvZ/OmpR system modulates the expression of numerous genes in response to environmental changes such as altered osmolarity, pH and nutrient content [57,58,59,60,61,62]. The transcriptional regulator OmpR has been best studied for its role in the inverse osmoregulation of the outer membrane porins OmpC and OmpF in *E. coli*. This is achieved by the binding of this factor, with different affinity, to multiple OmpR-binding sites within the regulatory regions of the *ompC* and *ompF* genes [54,56]. A large body of research has demonstrated that OmpR influences a wide variety of other cellular processes in *E. coli* [63,64,65,66], enteropathogenic *Salmonella* and *Shigella* [67,68], as well as pathogenic species of *Yersinia* [69,70,71]. The relationship between virulence and the activity of the OmpR protein has been described for *Y. enterocolitica* [72,73,74,75]. These studies showed that *Y. enterocolitica* OmpR can serve a variety of functions that are often specific to this organism, which highlights the pleiotropic role of this regulatory protein [76,77,78]. The results of studies in *Y. enterocolitica* emphasize the importance of OmpR in controlling the expression of several transcriptional regulators. The expression of FlhDC, the master regulator of motility genes, appears to be positively regulated by OmpR in *Y. enterocolitica* [79], which is in contrast to the negative role played by OmpR in *E. coli* [64]. The same positive regulation of *flhDC* by OmpR has also been demonstrated in *Y. pseudotuberculosis* [70]. Marked inhibition of AcrR, a regulator of multidrug transporter gene expression, was also noted in *Y. enterocolitica* [77]. Finally, KdgR, a negative regulator of oligogalacturonide-uptake genes in *Y. enterocolitica*, was identified as a direct target for positive OmpR-dependent regulation [78]. Thus, OmpR is involved in the control of diverse responses to environmental changes by altering the expression of downstream transcriptional regulators and functions as a global regulator in *Y. enterocolitica*.

Recently, differential proteomic analysis of *Y. enterocolitica* strain Ye9 (bio-serotype 2/O:9) revealed the role of OmpR in modulating (positively or negatively) the abundance of many proteins, including three iron transport system receptors: FepA for the uptake of ferrienterobactin, FecA for ferric dicitrate and HemR for heme compounds [80]. These results prompted us to investigate the link between OmpR and iron acquisition in *Y. enterocolitica*. It seemed reasonable to hypothesize that OmpR might influence the expression of iron-regulated genes by modulating expression of the Fur regulator. Thus, we investigated the role of OmpR in *fur* expression and examined the influence of diverse environmental factors on OmpR-dependent *fur* regulation. Subsequently, we determined whether OmpR can influence *fur* transcription by direct binding to the *fur* regulatory region. Lastly, we examined how OmpR-dependent regulation of *fur* affects the expression of Fur regulon genes.

By the application of reporter gene transcriptional fusions and RT-qPCR analysis, we show that OmpR inhibits the transcription of *fur* in *Y. enterocolitica*. Among the environmental factors tested, only pH and osmolarity influenced *fur* expression, but irrespectively of the activity of OmpR. Electrophoretic mobility shift assays showed that OmpR inhibits *fur* expression through binding to specific motifs present in the *fur* regulatory region. Quantification of a Fur-FLAG hybrid protein by Western blotting confirmed that the level of the Fur protein is negatively regulated by OmpR. The inhibition of *fur* transcription by OmpR was also found to occur in *E. coli*. Moreover, we examined whether OmpR could affect the expression of the *fecA*, *fepA* and *feoA* genes, members of the *Y. enterocolitica* Fur regulon, encoding proteins that participate in the transport of ferric and ferrous iron into cells. Our data demonstrate that the transcription of these genes is subject to a complex mode of regulation involving the OmpR and Fur regulators.

## 2. Results

### 2.1. Characterization of the Fur Promoter Region in Y. enterocolitica Strain Ye9N

**Figure 1:**
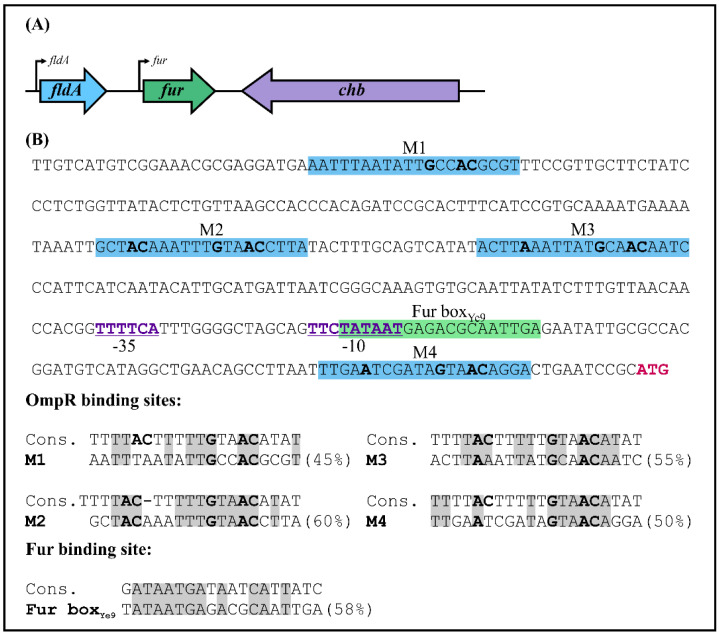
Chromosomal organization of the *Y. enterocolitica fur* locus. (**A**) Genes comprising the *Y. enterocolitica fur* locus. The directions of transcription are indicated by arrows. The encoded products are: *fur*—Fur (ferric uptake regulator); *fldA*—flavodoxin I; *chb*—chitobiase. (**B**) The *fur* promoter region of *Y. enterocolitica* Ye9N. The nucleotide sequence from −368 to +3 nt relative to the first nucleotide of the *fur* ATG start codon (colored pink) is shown. The −10 and −35 motifs of the *fur* promoter are marked purple, bold and underlined. The putative OmpR-binding sites (M1, M2, M3, M4) are highlighted by a blue background, while the potential Fur-binding site (Fur box_Ye9_) is highlighted by a green background. The putative OmpR- and Fur-binding sites are shown aligned to the *E. coli* consensus sequences (% identity values are shown). Identical nucleotides in the compared sequences are shaded gray. In both the promoter region sequence and alignments, nucleotides important for OmpR binding are in bold.

Analysis of the shotgun genome sequence of *Y. enterocolitica* strain Ye9N (2/O:9) (NCBI/GenBank Acc. No. NZ_JAALCX000000000.1) localized the *fur* gene, encoding the Fur repressor (an ORF of 447 nt, 148 aa), downstream of *fldA*, encoding flavodoxin I (175 aa), and upstream of *chb*, encoding chitobiase (891 aa) (Figure 1A). In silico analysis revealed that the *fldA-fur-chb* arrangement is highly conserved among *Y. enterocolitica* strains of different bio-serotypes and other pathogenic *Yersinia* species, i.e., *Y. pestis* and *Y. pseudotuberculosis* (Figure S1A). The *fldA* and *fur* ORFs are in the same transcriptional orientation, whereas *chb* is oriented divergently. The relatively long *fldA*-*fur* intergenic region (368 bp) suggests the potential for regulatory interactions. A putative promoter located upstream of *fur* (P*fur*) was identified using the software BPROM [81]. The −10 and −35 core promoter motifs are located 84 and 107 nt upstream of the start codon of *fur* and show partial resemblance to the respective consensus sequences for *E. coli* σ^70^ RNA polymerase (TATAAT and TTGACA) (Figure 1B). This analysis also revealed the presence of one putative Fur-binding site (Fur box_Ye9_, 5′-TATAATGAGACGCAATTGA-3′) in the *fur* promoter, overlapping the −10 sequence, with 58% identity to the *E. coli* Fur box consensus sequence, a 19-nt inverted repeat (5′-GATAATGATAATCATTATC-3′) [25]. Interestingly, four possible 20-nt OmpR-binding sites (OBSs) were also identified in the *Y. enterocolitica fur* promoter region (Figure 1B). These putative OBSs, named M1, M2, M3 and M4, are centered at −331, −227, −191 and −20 bp relative to the start codon of *fur* and, respectively, exhibit 45%, 60%, 55% and 50% identity to the *E. coli* consensus sequence proposed by Maeda et al. 1991 [82]. Moreover, two of four OBSs (M2 and M4) and a Fur box were revealed in the *fur* regulatory region of *Y. pestis* and *Y. pseudotuberculosis* (Figure S1B).

### 2.2. OmpR Inhibits the Activity of the Ye9 Fur Promoter 

**Figure 2:**
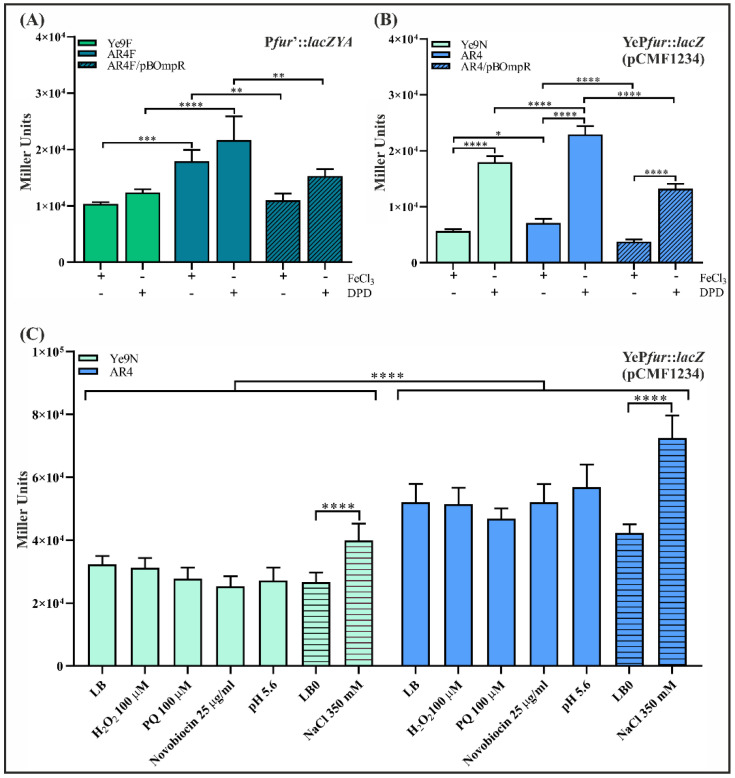
OmpR-dependent regulation of *fur* transcription in *Y. enterocolitica* Ye9N. (**A**) Expression of a chromosomally encoded P*fur*’::*lacZYA* transcriptional fusion in strains Ye9F (WT) and AR4F (Δ*ompR*) and complemented strain AR4F/pBOmpR (expresses OmpR from Ye9). β-Galactosidase activity was measured in strains grown to early stationary phase in LB medium at 26 °C, under iron-limiting (150 μM DPD) or iron-replete (10 μM FeCl_3_) conditions. (**B**) Expression of plasmid-encoded YeP*fur*::*lacZ* transcriptional fusion in strains Ye9N (WT) and AR4 (Δ*ompR*) and complemented strain AR4/pBOmpR (expresses OmpR from Ye9). β-Galactosidase activity was measured in strains grown in LB medium supplemented with FeCl_3_ or DPD, as above. (**C**) The influence of environmental factors on the expression of a YeP*fur*::*lacZ* transcriptional fusion encompassing the P*fur* region with four potential OmpR-binding motifs (pCMF1234) in strains Ye9N (WT) and AR4 (Δ*ompR*). The strains were grown to late exponential phase in LB (smooth bars) or LB0 (hatched bars) medium at 26 °C, then different stress factors were added before overnight incubation. The presented data (**A**–**C**) represent mean β-galactosidase activity values with SD from three independent experiments, each performed using at least triplicate cultures of each strain. Significance was calculated using one-way ANOVA (* *p* < 0.05, ** *p* < 0.01, *** *p* < 0.001, **** *p* < 0.0001).

In order to establish the role of OmpR in *fur* regulation, *Y. enterocolitica* strains Ye9F (WT) and AR4F (*ompR* deletion mutant) harboring a single-copy chromosomal P*fur’*::*lacZYA* transcriptional fusion were constructed using a mobilizable suicide plasmid. The Ye9 *fur* promoter region was cloned upstream of a promoterless *lacZYA* operon in vector pFUSE. The resulting plasmid pFUSE*fur* was transferred to strains Ye9N and AR4 by conjugation and exconjugants harboring plasmid co-integrates produced by a single crossover event between the *Y. enterocolitica* sequences present on the plasmid and the bacterial *fur* locus were selected. Correct integration was confirmed by PCR and sequencing (data not shown). β-galactosidase activity was measured in early stationary phase cultures of the resulting strains Ye9F and AR4F grown at 26 °C under iron-limiting (DPD, 150 µM) or iron-replete conditions (FeCl_3_, 10 µM). The results presented in Figure 2A demonstrate that β-galactosidase activity in the OmpR-deficient mutant AR4F was increased 1.7-fold compared to Ye9F, suggesting that *fur* transcription is repressed in an OmpR-dependent manner. The addition of iron chelator DPD to Ye9F cultures resulted in slightly higher reporter gene expression compared to those supplemented with FeCl_3_. However, a lack of response to iron availability was observed in the *ompR* mutant. To confirm the negative OmpR-dependent regulation of *fur*, plasmid pBOmpR carrying a gene coding for the His-tagged OmpR protein was used to complement the *ompR* mutation in strain AR4F. The β-galactosidase activity in AR4F/pOmpR was decreased 1.6-fold compared to AR4F (Figure 2A). The introduction of empty vector pBBR1 MCS-3 did not affect the *lacZ* expression level (data not shown). Thus, complementation of the *ompR* mutation resulted in the inhibition of *fur* expression, confirming that OmpR is required for the repression of *fur*.

The effect of OmpR on the expression of *fur* was also examined in the wild-type strain Ye9N and in the strain lacking *ompR* (AR4) by RT-qPCR (Figure S1). To determine whether iron influences *fur* transcript levels, RT-qPCR was performed using RNA isolated from the same strains grown under iron-limiting (DPD) and iron-replete (FeCl_3_) conditions. A visible (~2-fold) increase in the level of *fur* mRNA was detected in the strain lacking *ompR*. This corroborated the results of the *lacZ* reporter gene study showing OmpR-dependent inhibition of *fur*. In addition, *fur* transcript levels were not significantly changed by iron content (Figure S2). 

Finally, to confirm that OmpR affects *fur* expression through the regulation of *fur* promoter activity, we generated a transcriptional fusion of the Ye9 *fur* promoter region (YeP*fur*), containing four predicted OmpR-binding sequences, with the *lacZ* gene in vector pCM132Gm. The resulting plasmid pCMF1234 was introduced into strains Ye9N and AR4. Quantification of β-galactosidase activity along the bacterial growth curve showed that *fur* promoter activity in both strains increased around 2-fold in the early stationary phase compared with exponentially growing cells (Figure S3). The strains carrying pCMF1234 were grown to the early stationary phase at 26 °C in LB supplemented with FeCl_3_ (10 µM) or an iron chelator (DPD, 150 µM), then β-galactosidase activity was measured (Figure 2B). According to these measurements, the activity of YeP*fur* was slightly higher in the Δ*ompR* mutant strain compared to Ye9N in the ron-replete medium, and this difference was clearer under iron-limiting conditions. Complementation of the *ompR* mutation in strain AR4 by introducing plasmid pBOmpR led to a significantly reduced level of YeP*fur* activity, confirming the role of OmpR in the inhibition of Ye9 *fur* expression. Interestingly, in these experiments using the plasmid-encoded YeP*fur*::*lacZ* transcriptional fusion, we observed ~3-fold higher reporter activity in iron-limiting (DPD) than in iron-replete (FeCl_3_) conditions, for all studied strains (Figure 2B). 

### 2.3. Effect of Environmental Signals on OmpR-Dependent Ye9 Fur Expression 

The data presented above show that the expression of *fur* in *Y. enterocolitica* wild-type strain Ye9 is negatively regulated by OmpR and iron availability. The presence of a Fur-binding box in the *fur* promoter region (Figure 1) suggests that autoregulation of *fur* can occur. We next examined the OmpR-dependent regulation of *fur* expression under various environmental stresses: low pH, high osmolarity, novobiocin, paraquat and H_2_O_2_. It has been shown previously that OmpR modulates the expression of numerous genes in response to altered osmolarity and pH in *E. coli* and *Salmonella* [57,58,59,60,61,83]. In *Salmonella*, it was hypothesized that DNA relaxation due to the presence of novobiocin might promote OmpR binding to DNA to modulate expression of regulators SsrA and HilC encoded by the pathogenicity islands SPI-2 and SPI-1, respectively [84]. On the other hand, *E. coli fur* expression was shown to be responsive to oxidative stress generated by treatment with hydrogen peroxide or paraquat [47].

To determine the effects of these stresses on Ye9 *fur* expression, the plasmid-encoded YeP*fur*::*lacZ* transcriptional fusion (pCMF1234) was used. In order to attribute changes in *fur* expression to OmpR, these experiments were carried out using both the wild type (Ye9N/pCMF1234) and Δ*ompR* mutant (AR4/pCMF1234) grown in LB medium and in iron-limiting (150 µM DPD) conditions.

The addition of hydrogen peroxide (100 µM H_2_O_2_) or the superoxide generator paraquat (PQ, 100 µM) to the LB medium had no effect on *fur* expression in the strains Ye9N/pCMF1234 and AR4/pCMF1234 (Figure 2C). To ascertain whether the expression of *fur* responds to high osmolarity, both strains were grown in LB0 medium (10 g/L Tryptone, 5 g/L Yeast Extract) with or without the addition of 350 mM NaCl. High osmolarity appeared to slightly increase (~1.5-fold for Ye9N and ~1.7 for AR4) *fur* expression irrespective of the presence of OmpR (Figure 2C). We did not observe any changes in YeP*fur* activity in either strain grown in LB medium at low pH (pH 5.6). The presence of novobiocin, known to influence DNA topology in an OmpR-dependent manner [84], also had no effect on *fur* promoter activity in the studied strains (Figure 2C). No significant differences in *fur* expression were observed in response to the applied environmental stimuli in the absence of iron (data not shown). It is worth mentioning that during incubation of the tested strains under the studied conditions, i.e., in the presence of PQ, H_2_O_2_, novobiocin, low pH or altered osmolarity, we saw no significant differences in the growth of cultures.

### 2.4. Fur Abundance in Y. enterocolitica Is Modulated by the OmpR Regulator and Certain Environmental Conditions 

**Figure 3:**
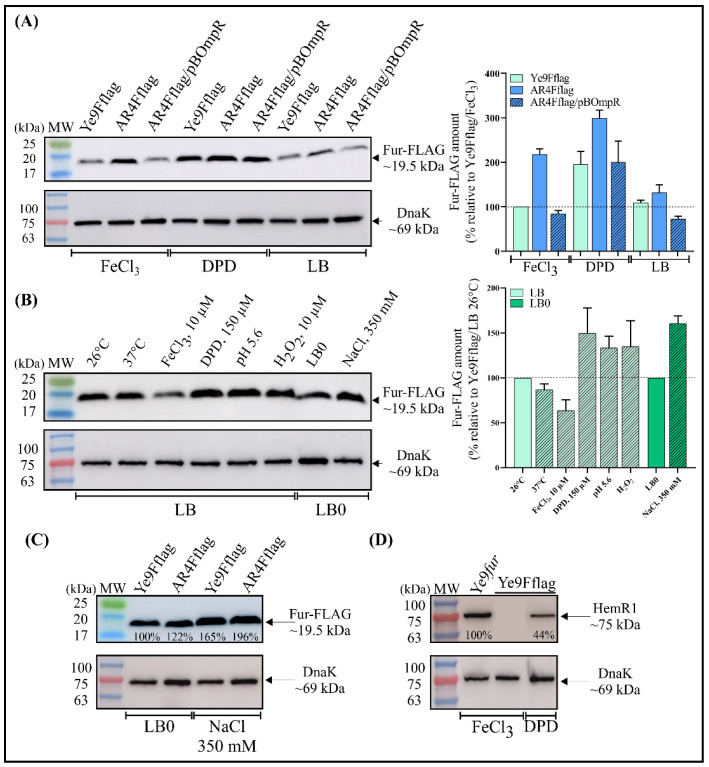
Western blot analysis of Fur expression in *Y. enterocolitica* Ye9N. (**A**–**C**) Levels of Fur-3×FLAG were analyzed in *Y. enterocolitica* cells grown in LB at 26 °C (unless indicated) for 24 h until stationary phase. Western blotting of cell extracts was performed with a monoclonal mouse anti-FLAG epitope antibody. As a loading control, the blots were also probed with an antiserum specific for cytoplasmic molecular chaperone DnaK. The Fur-FLAG band is ~19.5 kDa in size and DnaK ~69.0 kDa. (**A**, left panel) Comparison of Fur levels in the following strains: Ye9Fflag (Ye9N wild type with chromosomal *fur*::3×*flag* translational fusion), AR4Fflag (Δ*ompR* mutant AR4 with chromosomal *fur*::3×*flag* translational fusion), AR4Fflag/pBOmpR (AR4Fflag complemented with a plasmid carrying the wild-type *ompR* allele). The strains were grown in LB or in LB supplemented with FeCl_3_ (10 μM) or DPD (150 μM). (**A**, right panel) Quantification of the amount of Fur from three independent Western blots is shown as graphs with mean ± SEM. The amount of Fur measured in Ye9Fflag grown in LB + FeCl_3_ was set to 100%. (**B**, left panel) The strain Ye9Fflag was grown at a different temperature (26 vs. 37 °C), iron concentration (FeCl_3_ vs. DPD), reduced pH (5.6), increased oxygen stress (H_2_O_2_) or higher osmolarity (LB0 vs. LB0 plus 350 mM NaCl). (**B**, right panel) Quantification of the amount of Fur from three independent Western blots is shown as graphs with mean ± SEM. The amount of Fur measured in Ye9Fflag grown in LB at 26 °C was set to 100%. (**C**) *Y. enterocolitica* strains differing in OmpR content were grown at 26 °C in LB0 and in LB0 supplemented with 350 mM NaCl. The percentage values indicate the protein band intensities relative to Ye9Fflag grown in LB0. (**D**) Levels of HemR1 protein were analyzed in *Y. enterocolitica* cells grown in LB supplemented with FeCl_3_ or DPD at 26 °C for 24 h until stationary phase. Western blotting of cell extracts was performed with a polyclonal anti-HemR1 antibody (upper panel), and the blots were also probed with an anti-DnaK antibody as a loading control (lower panel). The HemR1 band is ~ 75.0 kDa in size. HemR1 levels were compared in strains Ye9Fflag and Ye9*fur* (*fur* deletion mutant of Ye9N, Δ*fur*::Gm). The percentage values indicate the protein band intensities relative to Ye9*fur*. The presented results (**A**–**D**) are representative of at least three independent experiments. The immunoreactive bands were quantified by densitometric analysis using ImageQuant TL analysis software (GE Healthcare). MW—3-color prestained protein marker (DNA Gdańsk).

To study the role of OmpR and iron content in modulating the abundance of the Fur protein, an in-frame translational fusion of Fur with a 3×FLAG epitope at the C-terminal end was constructed in the suicide vector pDS132. The obtained plasmid pDSFur-FLAG was introduced into the chromosome of Ye9N and its isogenic mutant AR4 (Δ*ompR*) by homologous recombination to replace the respective wild-type *fur* alleles. The correctness of the obtained fusions was confirmed by PCR and DNA sequencing (data not shown). These strains were grown in LB medium or LB supplemented with FeCl_3_ or DPD and the Fur-3×FLAG protein was detected in whole cell lysates by Western blotting using an anti-FLAG antibody (Figure 3A). The epitope-tagged Fur protein showed strong and specific reactivity with the anti-FLAG antibody. In the wild-type strain, the amount of Fur-3×FLAG increased by ~95% under iron-limiting conditions (LB + DPD), compared with iron-replete conditions (LB + FeCl_3_) or LB alone, confirming the regulatory role of iron in modulating the abundance of Fur. Fur-3×FLAG was present in higher amounts in the *ompR* deletion mutant (AR4Fflag) than in the wild-type Ye9Fflag strain, indicating that OmpR has an inhibitory effect on the level of the Fur protein (Figure 3A). However, the level of de-repression caused by the absence of OmpR varied according to the growth medium, i.e., a ~120% increase occurred in LB with FeCl_3_, ~100% in LB with DPD and only ~20% in LB medium. Complementation of the *ompR* mutation in the strain AR4Fflag with the plasmid pBOmpR resulted in a decrease in the amount of Fur-3×FLAG independently of the iron content of the medium, which is consistent with an inhibitory effect of OmpR on *fur* expression (Figure 3A). The Western blotting results were validated by probing the same blots with an antiserum specific for the cytoplasmic molecular chaperone DnaK to confirm equal protein loading. The intensity of the DnaK bands did not differ significantly between the tested samples.

We next examined the influence of different environmental factors (temperature, iron content, low pH, oxidative stress or high osmolarity) on Fur-3×FLAG synthesis in Ye9Fflag. As shown in Figure 3B, iron clearly reduced the abundance of Fur-3×FLAG, while a higher temperature (37 vs. 26 °C) had a slight inhibitory effect on Fur-3×FLAG synthesis. In contrast, low pH (5.6), oxidative stress caused by hydrogen peroxide (100 µM H_2_O_2_) and high osmolarity (350 mM NaCl) led to increased Fur production (Figure 3B).

The abundance of Fur-3×FLAG was then checked in strains differing in OmpR content, i.e., Ye9Fflag (WT) and AR4Fflag (Δ*ompR*), grown in LB0 and LB0 supplemented with NaCl (350 mM) (Figure 3C). Higher levels of Fur-3×FLAG were observed in the high-osmolarity medium (350 mM NaCl) regardless of the presence of OmpR, which suggested that osmoregulation occurs independently of this regulator.

Finally, to confirm the functionality of the Fur-3×FLAG fusion protein, expression of the outer membrane protein HemR1 was examined. It was shown previously that production of HemR1 in *Y. enterocolitica* is significantly influenced by iron and Fur content and is undetectable in wild-type strain Ye9N grown under iron-replete conditions and highly abundant in iron-depleted conditions [15]. Western blotting with an anti-HemR1 antibody showed an elevated level of HemR1 in the strain Ye9Fflag, synthesizing Fur-3×FLAG, grown under iron-limiting conditions, whereas no HemR1 could be observed in iron-replete conditions (Figure 3D). In strain Ye9N with the Δ*fur* mutation, HemR1 production was notably increased, confirming that Fur is involved in iron-dependent inhibition of HemR1 protein production. These data confirm the regulatory function of Fur/iron and show that the presence of the 3×FLAG peptide did not alter the activity of the Fur regulator.

### 2.5. The Effect of OmpR and Successive Deletion of the Fur Regulatory Region on the Transcriptional Activity of the YePfur Promoter

**Figure 4:**
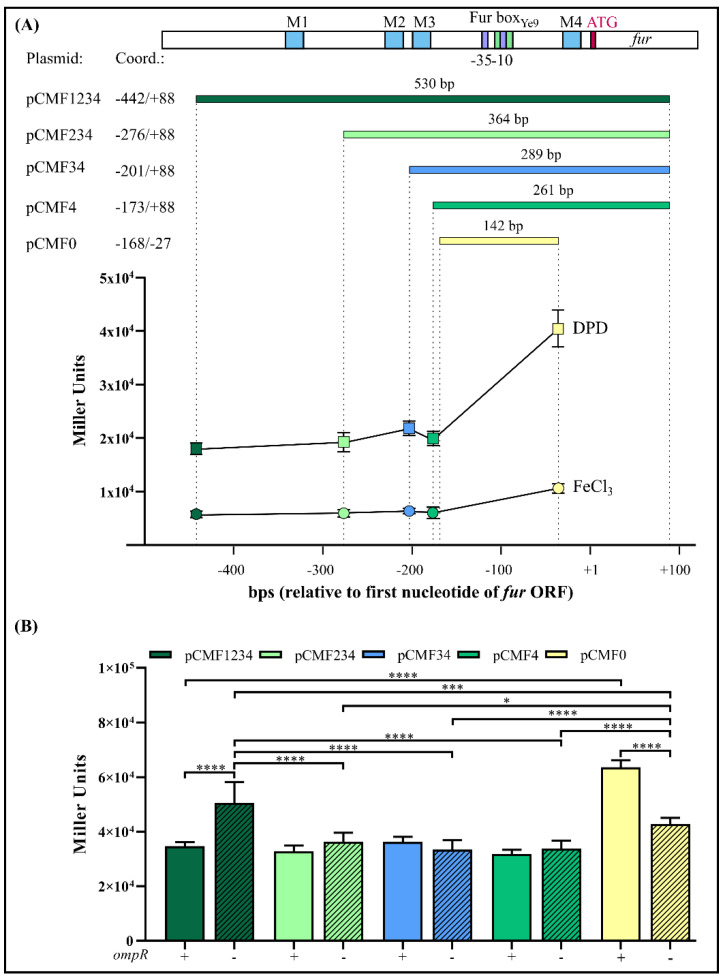
Effect of successive deletion of the *Y. enterocolitica* Ye9N *fur* regulatory region on the expression of *fur*. (**A**) At the top, a schematic presentation of the *Y*. *enterocolitica fur* promoter region (YeP*fur*) with the location of potential OmpR-binding motifs (M1, M2, M3 and M4) marked. Below this, the different fragments of YeP*fur* used to construct transcriptional fusions with *lacZ* in pCM132Gm are shown. The sizes of the fragments are given and coordinates assigned, with +1 corresponding to the first nucleotide of the *fur* ATG start codon. At the bottom, the expression of the P*fur*::*lacZ* transcriptional fusions from the following plasmids is plotted: pCMF1234, pCMF234, pCMF34, pCMF4 and pCMF0. β-Galactosidase activity was measured in Ye9N (WT) transformants grown to early stationary phase in LB medium at 26 °C, under iron-limiting (150 μM DPD) or iron-replete (10 μM FeCl_3_) conditions. Data represent mean activity values with SD from three independent experiments. The obtained differences are statistically significant with *p* < 0.0001. Significance was calculated using one-way ANOVA. (**B**) Expression of P*fur*::*lacZ* fusions encompassing different fragments of YeP*fur* in strains Ye9N (WT, +*ompR*, smooth bars) and AR4 (Δ*ompR* mutant, −*ompR*, hatched bars). β-Galactosidase activity was measured in the strains grown to early stationary phase in LB medium at 26 °C. The presented data represent mean β-galactosidase activity values with SD from three independent experiments, each performed using at least triplicate cultures of each strain. Significance was calculated using one-way ANOVA (* *p* < 0.05, *** *p* < 0.001, **** *p* < 0.0001).

The presence of four potential OmpR-binding sites within the *fur* regulatory region (M1, M2, M3, M4; Figure 4A) prompted us to investigate whether the activity of the *fur* promoter depends on these *cis*-acting sequences. The *fur* regulatory region encompassing the four putative OBSs (fragment F1234) was successively deleted upstream of the predicted core promoter motifs to produce DNA fragments of different lengths that harbor a decreasing number of OBSs, i.e., fragments F234 (M2, M3, M4), F34 (M3, M4) and F4 (M4). In addition, fragment F0, lacking all OBSs, was obtained by removing OBS M4 located downstream of the −10 core promoter motif. These fragments were used to create transcriptional fusions with the *lacZ* reporter gene in the vector pCM132Gm, producing constructs pCMF234, pCMF34, pCMF4 and pCMF0, respectively (Figure 4A). The four plasmids were introduced into the wild-type *Y*. *enterocolitica* Ye9N and these strains, together with Ye9N harboring plasmid pCMF1234, carrying the fragment encompassing all four OBSs, were used to study the transcriptional activity of *fur* in cells grown to the early stationary phase in iron-replete and iron-limiting conditions. Regardless of the number of OBSs, YeP*fur*::*lacZ* expression was always higher under iron-limiting conditions, which indicated that all studied regulatory fragments contain *cis*-acting sequences responsive to iron. Since all transcriptional fusions retained the Fur-box sequence, this suggested that the regulator Fur was responsible for the observed iron-dependent repression. However, sequential deletion of three putative OBSs from the distal 5′-end of the *fur* regulatory region (M1, M2, M3) did not significantly affect the expression of YeP*fur*::*lacZ*. Interestingly, shortening of the *fur* regulatory region by removal of OBS M4, located downstream of the −10 promoter motif (i.e., construct pCMF0), led to a ~50% increase in β-galactosidase activity in both iron-replete and iron-limiting conditions, which suggests some role for this *cis*-regulatory sequence in the inhibition of YeP*fur* activity (Figure 4A).

To elucidate the role of OmpR in the regulation of the *fur* promoter, the YeP*fur*::*lacZ* transcriptional fusions described above were analyzed in the wild-type strain Ye9N and the isogenic Δ*ompR* mutant AR4 (Figure 4B), grown to the early stationary phase in LB medium and LB with DPD. As was previously shown, expression of the YeP*fur*::*lacZ* fusion with all four OmpR-binding sites present (i.e., fragment F1234) was significantly higher in the mutant AR4, lacking OmpR, compared with the wild-type strain Ye9N, both grown in LB medium. However, when the promoter region was deprived of the regulatory fragment containing OBS M1, no influence of OmpR on the expression of the YeP*fur*::*lacZ* fusion was observed. Moreover, further successive deletion of the *fur* regulatory region harboring M2 and M3 in the constructed fusions did not lead to changes in β-galactosidase levels in the *ompR*^+^ compared to the *ompR*^-^ background. Interestingly, when the *fur* regulatory region fused to *lacZ*-lacked sequences downstream of the −10 core promoter motif including the potential OBS M4, upregulation of expression was observed in strain Ye9N compared to the other fusions. However, less upregulation was noted in the Δ*ompR* mutant strain carrying the same fusion. Considering the transcriptional activity of the different YeP*fur*::*lacZ* fusions, OmpR-mediated repression of *fur* was most affected by deletion of putative OBS M4. Interestingly, the same pattern of OmpR-dependent expression of *fur*, determined using reporter gene fusions, was observed in cells grown under iron-limiting conditions (data not shown). Taken together, these results suggest that *fur* repression is mediated by iron/Fur and the OmpR regulator, which probably primarily interacts with site M4. The presence of iron does not appear to influence OmpR-dependent *fur* regulation.

### 2.6. Interaction of OmpR with the Full Length and Truncated Fur Regulatory Regions of Y. enterocolitica

**Figure 5:**
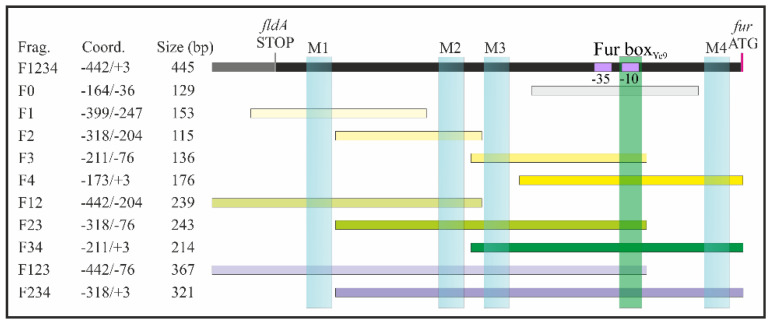
DNA fragments of the *Y. enterocolitica* Ye9N *fur* regulatory region used in electrophoretic mobility shift assay (EMSA) experiments. The position of DNA fragments within the extended *Y. enterocolitica fur* regulatory region is shown. The fragments contain one (yellow bars), two (green bars), three (purple bars) or four (black bar) putative OmpR-binding sites, or no sites (gray bar). Light blue rectangles indicate the location of the four potential OmpR-binding motifs M1, M2, M3 and M4. The putative Fur box_Ye9_ is marked in green, and the −10 and −35 core promoter motifs are marked in violet. The DNA fragment names, sizes and coordinates relative to the first nucleotide of the *fur* ATG start codon (marked in pink) are given. All fragments were amplified with primers listed in Supplementary Table S2.

**Figure 6:**
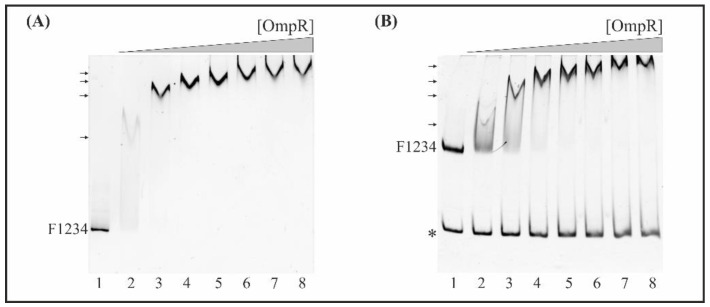
EMSA of the interaction between OmpR-His_6_ with the F1234 DNA fragment encompassing the whole *fur* regulatory region including four putative OmpR-binding sites. (**A**) Quantitative EMSA. The 445-bp *fur* promoter fragment F1234 was incubated without protein (lane 1) or with increasing amounts of purified OmpR: 0.65 (lane 2), 1.3 (lane 3), 1.95 (lane 4), 2.6 (lane 5), 3.25 (lane 6), 3.9 (lane 7), 4.55 μM (lane 8). (**B**) Competitive EMSA. Mixtures of the 445-bp *fur* promoter fragment F1234 and a 304-bp fragment of 16S rDNA (*) were incubated with increasing amounts of purified OmpR as in (**A**). The OmpR/DNA complexes were resolved on a native polyacrylamide gel and detected by SYBR Green staining. Shifted bands containing the *fur* fragment F1234 are marked by arrows.

The four potential OmpR-binding sites in the *fur* promoter region of *Y. enterocolitica* indicated by in silico analysis (M1, M2, M3, M4) display 45%, 60%, 55% and 50% identity to the *E. coli* consensus OmpR-binding site, respectively (Figure 1B). To determine whether these OBSs function as binding sites for OmpR in vitro, we performed electrophoretic mobility shift assays (EMSAs) using native polyacrylamide gels to detect binding of the purified OmpR-His_6_ protein to *fur* regulatory region DNA fragments containing different numbers of potential OmpR target sequences (Figure 5). First, EMSAs were performed using the 445-bp F1234 fragment encompassing the whole *fur* regulatory region (−442 to +3). This fragment, containing four potential OBSs (M1, M2, M3, M4) (Figure 6A), was PCR-amplified with the primer pair EFurYe1/EFurYe2 (Table S2) and incubated with increasing amounts of the His-tagged OmpR protein. Four shifted bands of decreasing mobility were observed as the OmpR concentration was increased from 0.65 to 3.25 µM, indicating the presence of multiple OmpR-binding sites. Next, a competitive bandshift assay was performed to study the ability of OmpR to alter the electrophoretic mobility of fragment F1234 when a control 304-bp 16S rDNA fragment was included in the binding reaction (Figure 6B). Again, slower migrating OmpR/DNA complexes were observed in the presence of OmpR at concentrations of 0.65 μM and above. OmpR had clearly bound to the *fur* promoter fragment encompassing four putative OBSs but failed to interact with the 16S rDNA fragment used as a negative control.

**Figure 7:**
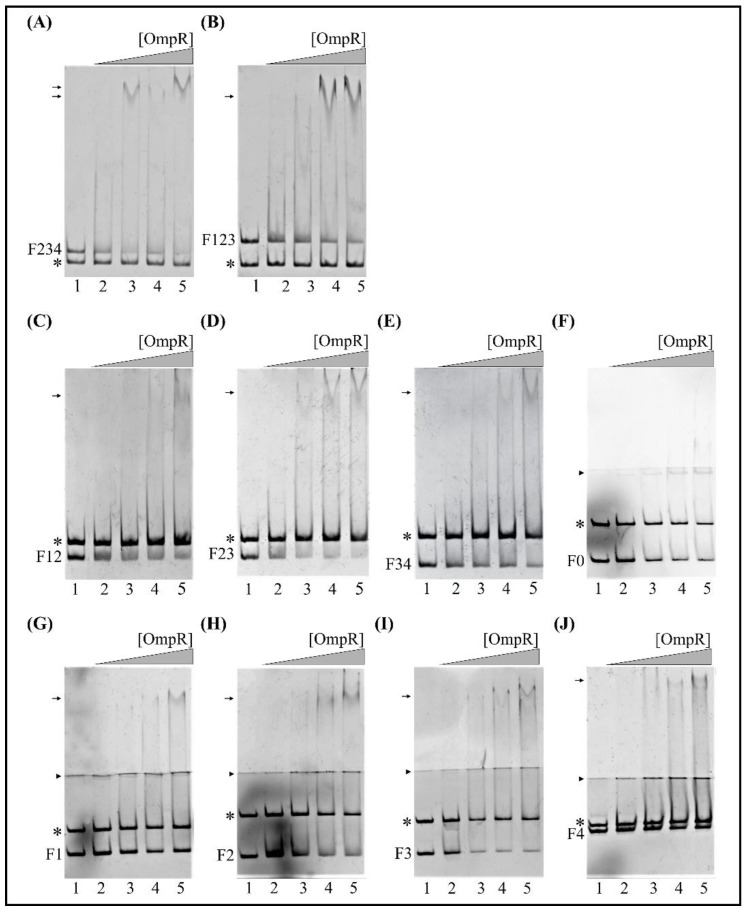
EMSAs of the interaction between OmpR-His_6_ and sub-fragments of the *fur* regulatory region carrying different numbers of potential OmpR-binding sites. (**A**,**B**) EMSAs with *fur* regulatory region DNA fragments comprising three potential OmpR-binding sites (OBSs): (**A**) lacking motif M1 from the 5′-end (F234, 321 bp) and (**B**) lacking motif M4 from the 3′-end (F123, 367 bp). (**C**–**E**) EMSAs with DNA fragments comprising two potential OBSs: (**C**) F12 (239 bp), (**D**) F23 (243 bp) and (**E**) F34 (214 bp). (**F**) EMSA with a DNA fragment lacking all OBSs. (**G**–**J**) EMSAs with DNA fragments containing one OBS: (**G**) F1 (153 bp), (**H**) F2 (115 bp), (**I**) F3 (136 bp) and (**J**) F4 (176 bp). In all cases, the DNA fragments were incubated without protein (lane 1) or with 0.65 (lane 2), 1.3 (lane 3), 1.95 (lane 4) or 2.6 μM (lane 5) OmpR-His_6_. Free DNA and protein/DNA complexes (arrows) are indicated. Fragments of 16S rDNA (*) were included in the reaction mixtures with the *fur* regulatory region fragments and served as a negative control. The OmpR/DNA complexes were resolved on a native polyacrylamide gel and detected by SYBR Green staining. In the EMSAs with very short DNA fragments (**F**–**J**), electrophoresis was performed on native polyacrylamide gels with two layers, i.e., a large pore 4.2% gel on the top and small pore 8.4% gel on the bottom, for better visualization of shifted complexes (arrowheads indicate the interface between the two gel concentrations).

Subsequently, EMSAs were performed using fragments of the *fur* regulatory region lacking OBS M1 or M4, located at the 5′- and 3′-ends of the sequence, respectively. These assays revealed that fragment F234 (−318 to +3), lacking OBS M1 (Figure 7A), and fragment F123 (−442 to −76), lacking M4 (Figure 7B), possessed OmpR-binding ability. However, in both cases, a smaller number of step-shifted slower migrating OmpR/DNA complexes appeared in the presence of OmpR at a higher concentration (1.31 μM), compared to fragment F1234 (−442 to +3) (Figure 6). The 304-bp 16S rDNA, used as the negative control in both cases, was not shifted. These data suggest that both M1 and M4 are responsible for the gel mobility shift pattern observed with the fragment F1234. 

Next, we performed EMSAs to study the ability of OmpR to directly interact with the two potential OBSs within fragments F12 (−442 to −204, M1 and M2), F23 (−318 to −76, M2 and M3) and F34 (−211 to +3, M3 and M4), in the presence of the 304-bp 16S rDNA control fragment. Native polyacrylamide gel electrophoresis of binding reactions containing equimolar amounts of these DNA fragments demonstrated the formation of slower migrating OmpR/F12, OmpR/F23 and OmpR/F34 complexes (Figure 7C–E). The complex OmpR/F23 was already present at a protein concentration of 1.3 μM (Figure 7D, lane 3). All of these OmpR/DNA complexes were specific, and their appearance was associated with the simultaneous disappearance of fragments F12, F23 and F34. OmpR was unable to bind the 16S rDNA negative control fragment. Notably, the 129 bp fragment F0 (−164 to −36), lacking all predicted OBSs, was not shifted at any of the OmpR concentrations employed (Figure 7F).

Finally, to determine the comparative hierarchy of OmpR binding by the putative OBSs, we tested their individual contributions to the binding of OmpR to the *fur* regulatory region. For this purpose, DNA fragments of ~100 to 200 bp long carrying single OBSs were used in EMSAs with increasing amounts of OmpR. For improved visualization of slower migrating OmpR/DNA complexes, electrophoresis was performed on native polyacrylamide gels cast in two layers, with a large pore gel on the top and a small pore gel on the bottom. 

The 153-bp DNA fragment F1 (−399 to −247), containing the most upstream *fur* regulatory region including OBS M1, exhibited a mobility shift at 2.6 µM OmpR (Figure 7G, lane 5). A lower concentration of OmpR (1.95 µM) started to shift the 115-bp fragment F2 (−318 to −204) encompassing OBS M2 (Figure 7H, lane 4), and the 136-bp fragment F3 (−211 to −76) containing M3 (Figure 7I, lane 4). In turn, the 176-bp fragment F4 (−173 to +3) containing OBS M4, located downstream of the *fur* core promoter, was shifted at 2.6 µM OmpR (Figure 7J, lane 5). The 211-bp 16S rDNA control fragment, present in each reaction together with the studied targets, was not shifted. Analysis of the EMSA results for these single OBS target fragments established the binding hierarchy as M2 and M3 > M1 and M4.

### 2.7. E. coli Fur Expression Is Negatively Regulated by OmpR

**Figure 8:**
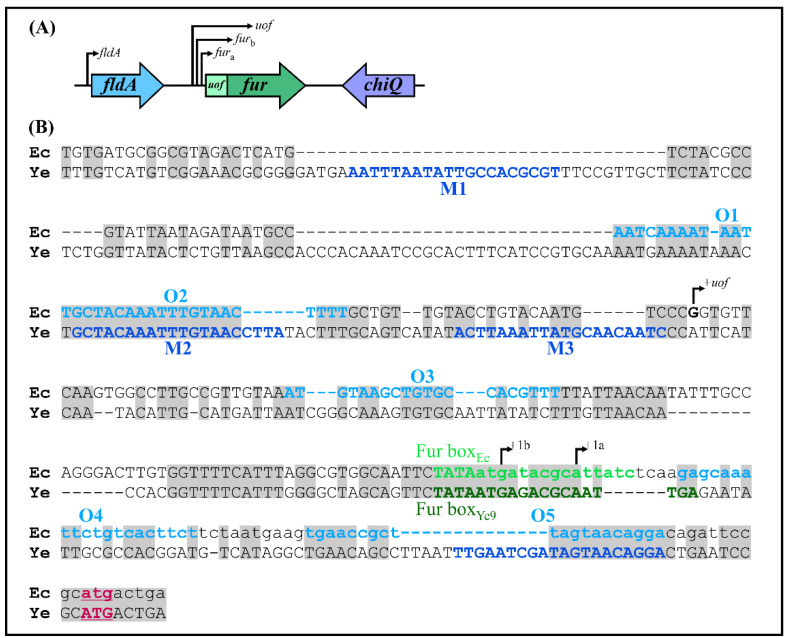
Comparison of the *fur* loci of *Y. enterocolitica and E. coli*. (**A**) Chromosomal organization of the *E. coli* *fur* locus. The direction of transcription from the *fldA*, *uof* and the two *fur* promoters is indicated by arrows (based on 47 and 85). The following products are encoded by these genes: *fldA*—flavodoxin I, *uof*—Fur leader peptide, *fur*—Fur (ferric uptake regulator), *chiQ*—chitosugar-induced lipoprotein. (**B**) Alignment of the *fur* regulatory regions of *E. coli* and *Y. enterocolitica***.** Chromosomal sequences of *E. coli* (Ec) and *Y. enterocolitica* (Ye) extending from the end of the *fldA* gene to the start codon of *fur* (plus five nucleotides from the end of the *uof* gene present in *E. coli*) were aligned using BlastN. Identical nucleotides are shaded gray. The putative transcription start sites of *E. coli fur* (a and b) and *uof* are indicated by +la, + lb and +1*uof*, respectively. Putative OmpR-binding sites (M1–M4) from *Y. enterocolitica* are in dark blue text, while putative *E*. *coli* OmpR-binding sites (O1–O5) are in light blue. The Fur-binding sites (Fur _boxEc_ and Fur _boxYe9_) are in light and dark green text, respectively. The sequence of *E. coli uof* is written in lowercase text. The *fur* translation start codons are in bold, underlined and colored pink.

In light of the results obtained for *Y*. *enterocolitica*, we decided to examine the regulatory region of *E. coli fur* for the presence of potential OmpR-binding sites. In silico analysis revealed that the organization of *fur* in *E*. *coli* K-12 strain MG1655 (GenBank Acc. No. NZ_CP009273.1) is similar to that of *Y*. *enterocolitica* Ye9N (GenBank Acc. No. NZ_JAALCX000000000.1), but with slight differences (Figure 8A). Immediately upstream of *E*. *coli fur*, there is the ORF *uof* encoding a short leader polypeptide, which is absent in *Y. enterocolitica*. Upstream of *uof*, there is the *fldA* ORF, as is also the case in the *Y. enterocolitca fur* locus. Downstream of *E. coli fur*, the *chiQ* ORF encoding chitosugar-induced lipoprotein is present instead of *chb*, encoding chitobiase in *Y. enterocolitica*. The *fur* gene of *E. coli* may be co-transcribed with *fldA* or with *uof* from their respective promoters. In addition, two *fur* gene promoters are recognized (*fur*a and *fur*b), so potentially four transcriptional units carrying *fur* might be induced under certain growth conditions (Figure 8A) [47,85,86].

**Figure 9:**
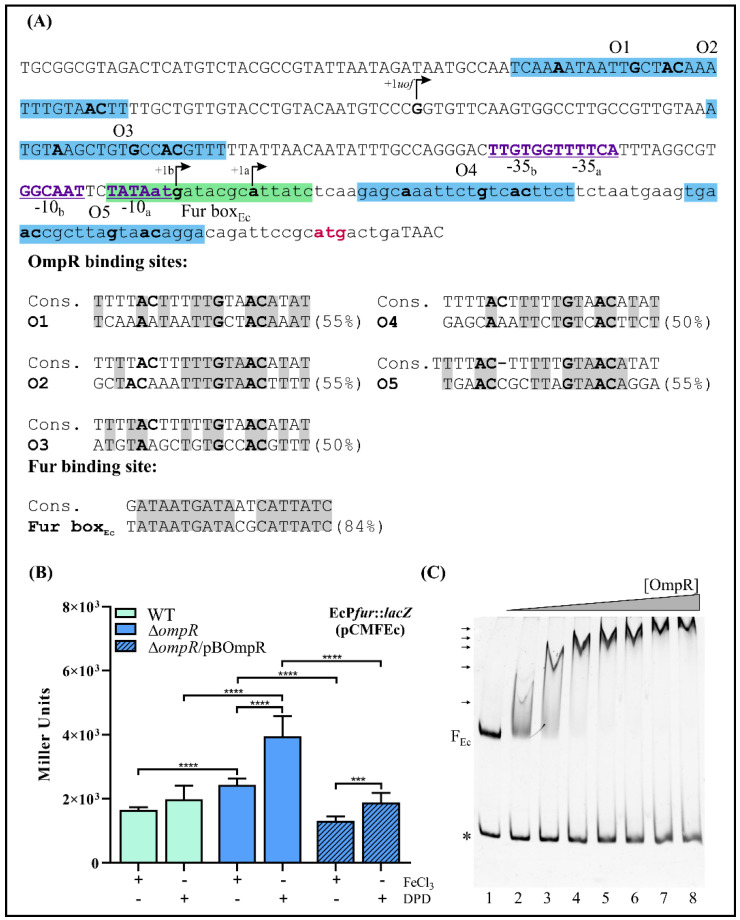
OmpR-dependent *fur* regulation in *E. coli*. (**A**) The *fur* promoter region of *E. coli* (based on 47 and 85). The nucleotide sequence from nt −284 to +12 relative to the first nucleotide of the *fur* ATG start codon. The putative OmpR-binding sites are highlighted by a blue background, while the Fur-binding site (Fur box_Ec_) is highlighted by a green background. The translation start codon of *fur* is colored pink. The −10 and −35 elements of the *fur* promoter are in bold, underlined and colored purple. The transcription start sites of *E. coli fur* (a and b) and *uof* are indicated by +la, +lb and +1*uof*, respectively. The lowercase sequence represents the *uof* ORF. The putative OmpR- and Fur-binding sites are shown aligned to the *E. coli* consensus sequences (% identity values are shown). Identical nucleotides in the compared sequences are shaded gray. In both the promoter region sequence and alignments, the nucleotides important for OmpR binding are in bold. (**B**) Expression of a plasmid-encoded EcP*fur*::*lacZ* transcriptional fusion (pCMFEc) in *E. coli* strains BW25113 (WT) and JW3368-1 (Δ*ompR*) and complemented strain JW3368-1/pBOmpR (expresses OmpR from *Y. enterocolitica* Ye9). β-Galactosidase activity was measured in strains grown at 37 °C to early stationary phase in LB medium, under iron-limiting (150 μM DPD) or iron-replete (10 μM FeCl_3_) conditions. The presented data represent mean activity values with SD from three independent experiments carried out in triplicate. Significance was calculated using one-way ANOVA (*** *p* < 0.001, **** *p* < 0.0001). (**C**) EMSA of the interaction between *Y. enterocolitica* OmpR-His_6_ and the *E*. *coli fur* promoter region (F_Ec_) depicted in Figure 9A. The 304-bp *fur* promoter fragment F_Ec_ and the 211-bp fragment of 16S rDNA (*, from *Y. enterocolitica* Ye9) were incubated together in reaction mixtures without protein (lane 1) or with an increasing amount of purified OmpR: 0.65 (lane 2), 1.3 (lane 3), 1.95 (lane 4), 2.6 (lane 5), 3.25 (lane 6), 3.9 (lane 7), 4.55 μM (lane 8). The OmpR/DNA complexes were resolved on a native polyacrylamide gel and detected by SYBR Green staining. Shifted bands containing the *fur* fragment F_Ec_ are marked by arrows.

DNA sequence alignment revealed that the *fur* promoter regions of *E. coli* (289 bp) and *Y. enterocolitica* (376 bp) are 86% identical, with multiple nucleotide deletions in the former sequence (Figure 8B). Five putative OmpR-binding sites O1, O2, O3, O4 and O5, respectively, exhibiting 55, 55, 50, 50 and 55% identity to the *E. coli* consensus, are present in the *E. coli fur* regulatory region (Figure 9A), suggesting some influence of OmpR on *fur* expression in this bacterium. Two of the OBSs, O1 and O2, are positioned upstream of the *uof* transcription start site (+1*uof*), whereas O3 is located upstream of the *fur* transcription start sites (+1*fur*_a_ and +1*fur*_b_) and O4 and O5 downstream of them (the OBSs O3, O4 and O5 are within the *uof* ORF). The Fur box overlaps the P*fur*_a_ −10 core promoter motif.

This in silico analysis and the results obtained for *Y*. *enterocolitica* prompted us to check the OmpR-dependent regulation of *fur* expression in *E*. *coli*. The EcP*fur*::*lacZ* transcriptional fusion in vector pCM132Gm was constructed similarly to the YeP*fur*::*lacZ* fusion. Briefly, a 250-bp DNA fragment comprising 242 bp upstream of the *fur* start codon plus 8 bp of the *fur* ORF (encompassing all five putative OmpR-binding sites) was cloned in pCM132Gm. The obtained plasmid pCMFEc was introduced into the wild-type strain BW25113 and *ompR* deletion mutant JW3368-1. These strains were grown at 37 °C in LB supplemented with FeCl_3_ (10 µM) or DPD (150 µM). β-Galactosidase assays performed on cells in the early stationary phase showed that expression from the *E*. *coli fur* promoter was ~1.5-fold higher in the Δ*ompR* mutant than in the WT strain in iron-replete conditions and ~2-fold higher in iron-limiting conditions (Figure 9B). Since the predicted amino acid sequences of the OmpR regulators of *Y*. *enterocolitica* and *E*. *coli* share 98% identity (data not shown), this led us to use OmpR of *Y. enterocolitica* to complement the Δ*ompR* mutation in *E. coli*. When OmpR was expressed from plasmid pBOmpR in the *E. coli* Δ*ompR* mutant background, *fur* promoter activity was decreased to the wild-type level. This confirmed the negative role of OmpR in *fur* regulation and showed that *Y. enterocolitica* OmpR can functionally substitute the OmpR of *E. coli* (Figure 9B). The presence of iron had no influence on EcP*fur*::*lacZ* expression in the wild-type strain, which contrasts with the clear effect in the Δ*ompR* mutant strain, under the tested conditions. Finally, to reveal whether OmpR interacts with the *fur* regulatory region of *E. coli*, we performed EMSAs using a DNA fragment containing all predicted OBSs (O1, O2, O3, O4, O5). Increasing amounts of *Y. enterocolitica* OmpR-His_6_ were incubated with the 304-bp *E. coli fur* regulatory region fragment (F_Ec_) and a 211-bp fragment of *Y. enterocolitica* Ye9N 16S rDNA was included in the binding reactions as a negative control. As shown in Figure 9C (lane 2), OmpR/DNA complexes of decreasing mobility were observed on a native polyacrylamide gel with an increasing concentration of OmpR, starting from 0.65 μM. Multiple shifted bands were observed as the OmpR concentration was increased up to 3.9 µM, suggesting that OmpR interacts with several sites within the *fur* regulatory region of *E. coli*.

### 2.8. Effect of OmpR on the Expression of Selected Fur Regulon Members in Y. enterocolitica

The next step in our studies was to examine whether the OmpR-dependent regulation of Fur might affect the expression of Fur regulon genes in *Y. enterocolitica.* Several genes belonging to the Fur regulon were chosen based on global analyses of iron assimilation and *fur* regulation performed previously in *Y. pestis* and *E. coli* [24,41,42] and a differential proteomic analysis of OmpR-regulated proteins in *Y. enterocolitica* [80]. Three genes controlled by Fur were selected: *fecA* and *fepA* encoding OM receptor proteins FecA and FepA necessary for transport of ferric dicitrate and ferrienterobactin, respectively, and *feoA* encoding an inner membrane protein required for Fe^2+^ transport (FeoABC system).

**Figure 10:**
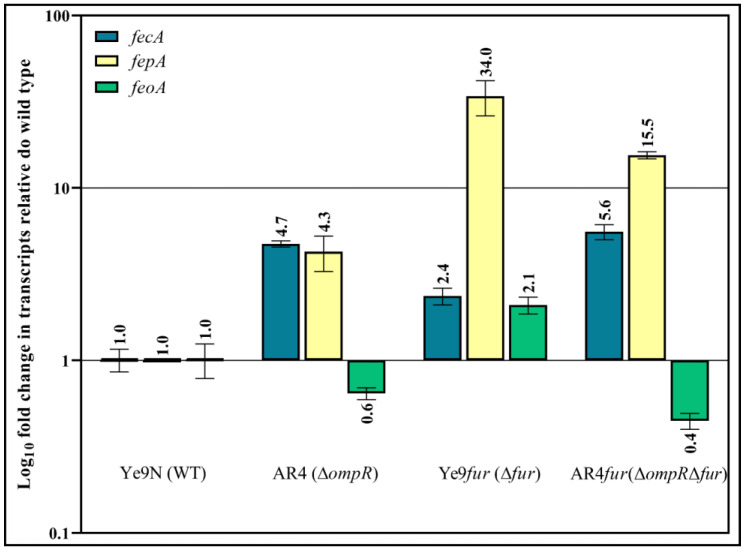
OmpR- and Fur-dependent regulation of *fecA, fepA* and *feoA* transcription in *Y. enterocolitica* Ye9N. Effect of OmpR on Fur regulon members expression determined by RT-qPCR. This analysis was performed using RNA prepared from cells of the wild-type strain (Ye9N), the Δ*ompR* mutant (AR4), the Δ*fur* mutant (Ye9*fur*) and the double Δ*fur*Δ*ompR* mutant (Ye9*furompR*), grown to early stationary phase in LB medium. Relative *fecA, fepA, feoA* and *ftnA* transcript levels, normalized to the amount of 16S rRNA, are shown, taking the mRNA level in Ye9N as 1. The mean value and SD obtained from at least three independent experiments are indicated for each strain.

Initially, the levels of the *fecA, fepA* and *feoA* mRNAs were assessed by RT-qPCR in strains Ye9N (the wild-type strain), Ye9*fur* (Δ*fur*), AR4 (Δ*ompR*) and AR4*fur* (Δ*ompR*Δ*fur*). The Δ*fur*::Gm mutants of *Y. enterocolitica* Ye9N (Ye9*fur*) and AR4 (AR4*fur*) were constructed by homologous recombination as described previously [15]. The obtained data show an increase in the mRNA transcript level of 2.4-fold for *fecA* and 34-fold for *fepA* in the Ye9*fur* strain (Δ*fur* background) compared to the wild-type strain, indicating Fur-mediated repression of both genes (Figure 10). In strain AR4 (Δ*ompR* background), the *fecA* and *fepA* transcript levels were increased 4.7-fold and 4.3-fold, respectively, relative to the wild-type strain, indicating that OmpR has a negative impact on *fecA* and *fepA* expression. Interestingly, the upregulation of *fecA* in the Δ*ompR* background was 2-fold higher than that observed in the Δ*fur* background and did not really change when these mutations were combined (Δ*ompR*Δ*fur*), indicating that the inhibitory effect of OmpR exceeds the repressive activity of Fur and can be observed regardless of Fur (Figure 10). In contrast, the strain devoid of Fur (Δ*fur* background) exhibited 8-fold higher *fepA* expression than that lacking OmpR (Δ*ompR* background). However, the combination of both mutations (Δ*ompR*Δ*fur*) led to a 2-fold decrease in the *fepA* transcript level, suggesting that OmpR may have a positive impact on *fepA* expression in the absence of Fur (Figure 10). Regarding the expression of the third tested gene, a 2.1-fold increase in *feoA* transcripts was observed in a Δ*fur* background relative to the wild-type strain, indicating inhibition of *feoA* expression by Fur (Figure 10). In turn, the *feoA* mRNA level decreased slightly in the Δ*ompR* genetic background in comparison to the wild type, indicating a weak positive influence of OmpR. Interestingly, in the double mutant (Δ*ompR*Δ*fur*), a ~4-fold decrease in the *feoA* mRNA transcript relative to the Δ*fur* background was observed, resulting in levels equivalent to those in the Δ*ompR* background. Thus, it seems that upregulation of *feoA* by OmpR may abolish Fur repression.

**Figure 11:**
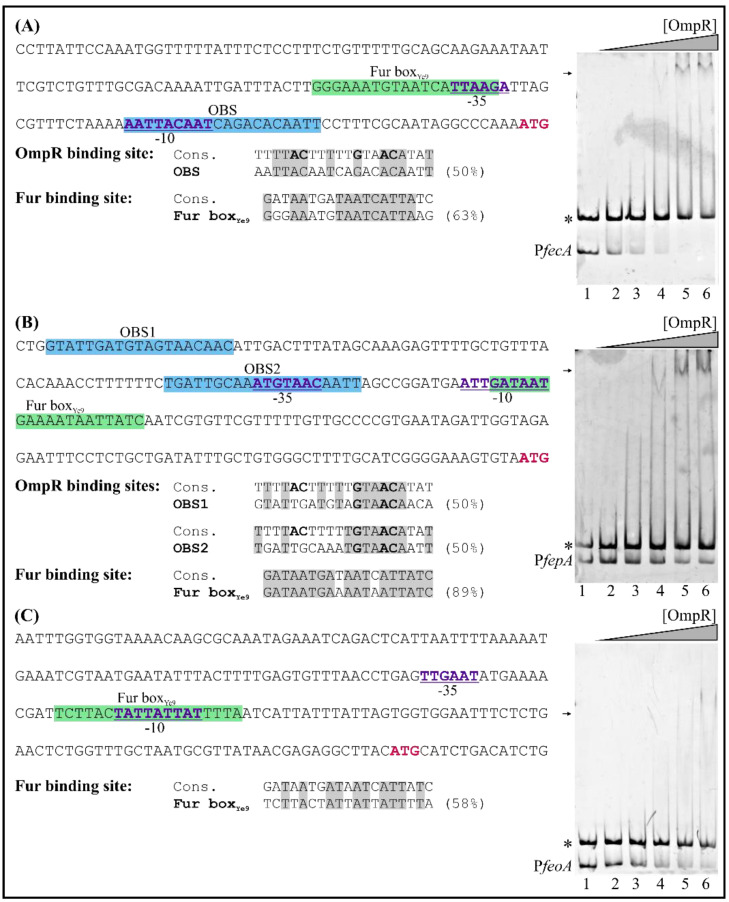
Binding of OmpR-His_6_ to the *fecA*, *fepA* and *feoA* regulatory regions examined using EMSAs. Left panels show the promoter of *fecA* (**A**) from −159 to +3 nt relative to the ATG start codon, *fepA* (**B**) from −213 to +3 nt relative to the ATG start codon and *feoA* (**C**) from −200 to +16 nt relative to the ATG start codon, while right panels are the results of EMSA experiments. The sequences shaded blue correspond to the putative OmpR-binding sites. The potential Fur-binding site (Fur box_Ye9_) is highlighted by a green background. The *fecA*, *fepA* and *feoA* start codons (ATG) are shown in bold and marked pink. Beneath the sequence, the putative binding sites are compared with the consensus OmpR and Fur-binding motifs of *E. coli*. The percentage identity to these sequences is shown. Identical nucleotides in the compared sequences are shaded gray. Nucleotides important for OmpR binding are in bold. The P*fecA* (189 bp), P*fepA* (247 bp) and P*feoA* (227 bp) promoter fragments and the 304-bp fragment of 16S rDNA (*, from *Y. enterocolitica* Ye9) were incubated together in reaction mixtures without protein (lane 1) or with an increasing amount of purified OmpR: 0.6 (lane 2), 1.2 (lane 3), 2.4 (lane 4), 4.8 (lane 5), 6 μM (lane 6). The OmpR/DNA complexes were resolved on a native polyacrylamide gel and detected by SYBR Green staining. Shifted bands containing the *fecA*/*fepA*/*feoA* promoter region are marked by arrows.

To clarify whether OmpR of *Y. enterocolitica* may participate in the direct regulation of *fecA*, *fepA* and *feoA* gene expression, we first analyzed their promoter regions in silico to identify putative OmpR-binding sites. One potential OBS was found in the promoter region of *fecA*, located at −40 to −21 nt relative to the start codon (overlapping the −10 core promoter motif) and two putative OBSs were identified in the promoter region of *fepA* (at −210 to −191 nt and −144 to −125 nt relative to the start codon) (Figure 11, appropriate left panels). Each OBS exhibits 50% identity to the consensus OmpR-binding site. We failed to detect any potential OBSs in the analyzed *feoA* promoter region. With regard to the Fur box, potential examples were identified in each of the three analyzed genes, overlapping the −10 or −35 core promoter motifs (Figure 11, left panels).

Regardless of the results of the in silico analysis, the promoter regions of the *fecA*, *fepA* and *feoA* genes were examined for OmpR binding using EMSAs. DNA fragments were incubated with increasing concentrations of OmpR (Figure 11, right panels). Shifted OmpR/DNA complexes were clearly produced when 189-bp *fecA* (−162 to +27 relative to the start codon) and 247-bp *fepA* (−305 to −59 relative to the start codon) fragments interacted with OmpR at a concentration of 2.4 µM (Figure 11, appropriate right panels). No shifting of an unrelated control DNA fragment (304-bp, 16S rDNA) was observed, confirming that these OmpR/DNA complexes were specific. In addition, their appearance was associated with the simultaneous disappearance of the *fecA* and *fepA* DNA fragments. The 227-bp *feoA* promoter region fragment (−195 to +32 relative to the start codon) interacted weakly with OmpR, and faint shifted OmpR/DNA complexes appeared at a concentration of 4.8 µM. Thus, this regulator appears to bind with low affinity to the studied *feoA* promoter region. Together, these EMSA results indicate that OmpR can interact directly with the *fecA* and *fepA* promoter regions in vitro, but the interaction with the *feoA* promoter seems to be much weaker.

## 3. Discussion

OmpR has been identified as a transcriptional regulator of various genes involved in the control of diverse cellular processes and functions in bacteria including *Y. enterocolitica* [58,68,75,87,88]. Based on the results of proteomic analyses, the production of a number of membrane proteins involved in the uptake and transport of compounds into *Y. enterocolitica* cells, including several that participate in iron or heme acquisition, is subject to control by the regulator OmpR [80]. The acquisition and storage of iron are modulated by the Fur regulator to maintain iron homeostasis necessary for bacterial survival [89]. In light of these findings, we initiated studies aimed at identifying any correlation between the function of the OmpR regulator and the expression of Fur in *Y. enterocolitica.* Despite the importance of Fur in the regulation of iron/heme acquisition in pathogenic *Yersinia* [8,9,15,41,90], the control of Fur biosynthesis is poorly understood. Analysis of the *fur* promoter region of *Y. enterocolitica* strain Ye9N identified one putative Fur-binding sequence and four predicted OmpR-binding sequences, suggesting a potential role for these regulators in the modulation of *fur* expression. In the course of this study, we identified OmpR as an inhibitor of *fur* expression, acting independently of Fur. Moreover, the activity of the YeP*fur* promoter was found to be weakly inhibited by iron, implying a negative autoregulatory role for Fur. The *fur* genes of other Gram-negative bacteria are often, but not always, negatively autoregulated in the presence of iron, due to the binding of Fur to the Fur-box motif [25,49,91,92]. However, the activity of the *fur* promoters of *Campylobacter jejuni* and *Burkholderia cepacia* was not influenced by iron availability [45,93].

To ascertain whether the *Y. enterocoltica* P*fur* is responsive to environmental cues, several factors known to influence OmpR and/or Fur level/activity in *E. coli* and *Salmonella* were tested [58,60,61]. OmpR is a component of the EnvZ/OmpR TCS, known to be one of the major regulatory systems in *E. coli* controlling the expression of outer membrane porins in response to osmolarity [55,94]. In an environment of high osmolarity, EnvZ phosphorylates the regulatory protein OmpR, leading to its activation and inverse regulation of the *E. coli ompC* and *ompF* genes [95]. In *Y. enterocolitica*, OmpR appears to be required for the inhibition of *fur* expression, so it might be expected that a decrease in the activity of YeP*fur* should be observed under high-osmolarity conditions. However, we found that *Y. enterocolitica fur* expression increases in conditions of high osmolarity and independently of the presence of OmpR. We presume that other mechanisms may be involved in the observed regulation, i.e., changes in DNA supercoiling and the activity of nucleoid structuring proteins such as H-NS, known to be involved in the osmoregulation of some genes in *E. coli* [96,97]. In addition, it has been shown that some genes of the OmpR regulon are not responsive to changes in osmolarity [98,99,100]. Interestingly, recent studies have provided evidence for the involvement of Fur as part of the complex circuit that controls the response to osmotic stress in halophilic bacteria [101]. Low pH was recognized as an environmental signal that influences the expression of several genes in an OmpR-dependent manner in many bacterial species [102,103,104]. However, low pH did not have a significant impact on YeP*fur* activity. We also showed that YeP*fur* activity is not influenced by novobiocin, a compound known to induce DNA relaxation. Changes in DNA topology may influence the activity of some promoters in an OmpR-dependent manner [84], but this does not seem to apply to YeP*fur*. An analysis of OmpR-dependent genes within Proteobacteria suggested that the OmpR regulon is not highly conserved across bacterial species and different regulatory networks exist to respond to the various environmental stress conditions encountered [105]. We observed no effect of hydrogen peroxide or the superoxide generator paraquat on YeP*fur* promoter activity. This is in contrast to the situation in *E. coli* where *fur* gene expression is activated by both stressors. Previously, OxyR was identified as a regulator that senses elevated levels of hydrogen peroxide to induce *fur* expression in *E. coli*, while SoxRS was found to increase *fur* expression in response to paraquat [47]. The absence of any observable effect of oxidative stress on the *fur* transcription of *Y. enterocolitica* parallels the results of studies investigating the *fur* genes of *B. cepacia*, *B. pseudomallei* and *P. aeruginosa* [93,106,107]. Since bacteria experience the continual fluctuation of numerous environmental parameters, which alters the expression of many genes including regulators, this would explain the difficulty in identifying a single factor mediating changes in the expression level of *fur*. When *Y. enterocolitica* passes from the external environment into the host body, conditions such as temperature, pH, iron and oxygen availability and osmolarity change comprehensively. Therefore, a combination of these factors might be responsible for modulating *fur* expression.

To clarify whether OmpR participates in the production of the Fur regulator, Western blot analysis was performed. The results show that OmpR reduces Fur protein levels, in agreement with the *fur* promoter activity test data. In addition, iron had a greater effect on the level of the Fur protein than on *fur* transcription, suggesting the presence of another regulatory mechanism affecting iron-dependent *fur* expression. The increase in Fur protein abundance produced by high osmolarity correlated with the upregulation of *fur* transcription under these conditions. Interestingly, low pH and H_2_O_2_ led to increased Fur synthesis that was not reflected at the transcriptional level. It has previously been shown that the effect of low pH on gene expression is complex and interconnected with other regulatory factors. Changes in mRNA abundance, and hence in the level of protein synthesis, may be the consequence of mRNA stabilization or destabilization [108]. Enhanced RNA stability at acidic pH, not only the RNA phosphodiester bond but also the aminoacyl-(t)RNA and peptide bonds, has been demonstrated [109]. Notably, the translation process and the regulation of mRNA stability are preferential targets of H_2_O_2_ in eukaryotic organisms [110].

Four potential OmpR-binding sites (M1–M4) were identified in the *fur* promoter region of *Y. enterocolitica*. These share considerable similarity with the OmpR consensus binding sequence defined based on sites identified within the *E. coli ompF* and *ompC* promoter regions [82]. The putative OmpR-binding sites in YeP*fur* share several features, in particular the central GXXAC motif shown to be critical for OmpR binding [56,111]. In the case of OBS M2, a conserved AC base pair located 9 nt away from the AC element of the central motif GXXAC was also present. This sequence configuration was previously demonstrated to promote the stability of OmpR binding [56,111]. Recently, by applying ChIP-chip analysis, SELEX-chip screening and ChIPMunk methods, other binding site motifs bound by OmpR with different affinities were identified in *E. coli* [58,105,112]. Thus, the available data indicate that OmpR has moderate binding sequence specificity.

The study of the relative contribution of four putative OmpR-binding sites on P*fur* activity revealed that deletion of the *fur* regulatory region from the 5′-end encompassing OBS M1, M2 and M3 did not affect the promoter activity. However, significant upregulation of *fur* occurred when the deletion was extended to include OBS M4, located downstream of the −10 core promoter motif, suggesting that M4 is most important for OmpR-dependent inhibition of *fur* expression. Unexpectedly, the extent of this upregulation of *fur* observed in the wild-type strain was reduced in the Δ*ompR* mutant background, concomitantly with the lack of OmpR, which suggests a role for other transcriptional regulators whose expression could be under OmpR control or some interplay with OmpR at the DNA sequence level. Upon analyzing other YeP*fur*::*lacZ* fusions in the absence of OmpR, we found that the removal of OBS M1, but not M2 and/or M3, led to the inhibition of *fur* expression, suggesting the presence of a *cis*-regulatory sequence for a putative transcriptional activator. In *E. coli*, the OxyR and SoxRS regulators co-activate *fur* expression in response to oxidative stress [47,105]. Our in silico analysis identified potential binding sites for these activators within the *Y. enterocolitica fur* promoter region. Between OBS M1 and M2, putative SoxR- and SoxS-binding motifs with 58% and 70% identity to the *E. coli* consensus sequences were revealed (data not shown) [105,113]. Moreover, an OxyR-binding site (OxBS) that overlaps OBS M2 was recognized (data not shown). The nucleotide sequence of *Y. enterocolitica* OxBS exhibits 100% identity to the *E. coli* OxBS sequence found in the *fur* promoter [47]. These potential regulatory sequences and OxyR and/or SoxRS might be responsible for activation of *fur* expression in *Y. enterocolitica*. However, confirmation of this hypothesis will require further detailed study, especially since the activation of *fur* expression by oxidative stress was not detected under the conditions tested. We cannot exclude the possibility that the observed effect of DNA sequence deletions on the expression from YeP*fur* is the result of changing the distance between regulatory sites, which alters the number of turns of the DNA helix, thus affecting the interaction of bound factors and influencing promoter activity, as has been demonstrated for the promoters of *ilvGp2* and the *araBAD* operon [114,115].

EMSAs were performed to demonstrate the ability of OmpR to bind to the *fur* promoter region in vitro and establish the OmpR-binding hierarchy of the OBSs. The analysis revealed that OBSs M2 and M3, with a higher degree of identity to the consensus binding site, were most important for OmpR binding in vitro. In addition, only M2 among the four OBSs has a second AC nucleotide doublet at the correct distance from the TGTAAC motif, recognized as the most highly conserved and critical sequence for OmpR binding [111]. The EMSA results allow us to establish the hierarchy of OmpR binding within the *fur* regulatory sequences as M2, M3 > M1, M4. Together, these data suggest that *fur* repression may depend on the presence of all four OBSs, which could bind OmpR molecules that interact with each other in a complex way. The regulation of *fur* expression by OmpR might be based on hierarchical and cooperative binding of this protein to OBSs in the *fur* regulatory sequence, as was observed previously for *ompC* and *ompF* of *E. coli* [116,117,118,119,120]. Progressive occupancy of the OBSs in the *fur* promoter region could provide differential modulation of the OmpR-mediated repression. The occurrence of multiple repressor binding sites has been described for OmpR in the *csgD* promoter [121] and for regulators CpxR [122] and CytR [123]. To summarize, our results show that OmpR inhibits *fur* expression by direct binding to YeP*fur* and that the other putative regulatory proteins may influence OmpR-mediated regulation to ensure the appropriate abundance of Fur in *Yersinia* cells.

In the light of our findings regarding OmpR and *fur* expression in *Y. enterocolitica*, we were curious whether OmpR participates in the regulation of *fur* in *E. coli*. Despite sequence divergence in the *fur* regulatory regions of *Y. enterocolitica* and *E. coli*, in silico analysis indicated five potential OmpR-binding sites in the latter. Experiments conducted with an EcP*fur*::*lacZ* transcriptional fusion confirmed that OmpR inhibits *fur* promoter activity in *E. coli*. EMSAs demonstrated in vitro binding of OmpR to an *E. coli fur* promoter region fragment encompassing all putative OBSs. The slower migrating stepshift OmpR/DNA complexes observed at higher OmpR concentrations confirmed the presence of more than one OBS. Interestingly, Genomic SELEX screening in *E. coli* identified a potential OmpR-binding site upstream of the *uof* sequence present in the *fur* regulatory region [112]. It is also noteworthy that microarray analysis has demonstrated the involvement of the EnvZ/OmpR system in the expression of a number of Fur-regulated genes in *E. coli*, particularly those concerned with enterobactin synthesis and transport [87].

Given the negative regulation of Fur by OmpR in *Y. enterocolitica*, we next examined whether OmpR could affect the expression of genes belonging to the *Y. enterocolitica* Fur regulon, involved in the transport of ferric and ferrous iron into cells. The genes selected for this analysis were *fecA* and *fepA*, encoding OM receptors of ferric siderophore-based iron transport systems, and *feoA* of the FeoABC system specific for ferrous iron transport, which have been characterized in both *E. coli* and *Yersinia* [89,124,125,126]. RT-qPCR data show that all three studied genes are repressed by Fur in *Y. enterocolitica* cells grown aerobically, which is in agreement with the results of studies in *E. coli* [18,127,128,129]. Interestingly, a recent study on the transcriptional activity of *feoABC* in *Y. pestis* revealed that Fur-mediated regulation of this locus occurs during microaerobic but not aerobic growth [20]. The most obvious transcriptional change (repression by Fur) was observed for the *fepA* gene encoding the FepA receptor for ferric enterobactin, the most potent siderophore of *E. coli* [130]. This may be due to the presence of a Fur box bearing most similarity to the 19-bp consensus sequence (a mismatch of only two base pairs compared to eight mismatches in the case of the Fur boxes of *fecA* and *feoA*), and hence strong Fur binding. Unexpectedly, we found that OmpR inhibits *fepA* and *fecA* expression, which appears at odds with the inhibition of their repressor Fur mediated by the same regulator. EMSAs revealed that OmpR is able to bind the promoter regions of *fecA* and *fepA*, suggesting that it directly influences their expression. Based on these results, we hypothesize that the negative regulation of both receptor genes by OmpR may overcome the modest positive effect of OmpR resulting from its inhibition of Fur. Interestingly, we observed a positive impact of OmpR on *fepA* expression in the absence of Fur, suggesting that additional regulatory mechanisms involving these two regulators may exist. It may be speculated that as yet unrecognized transcriptional or post-transcriptional regulatory mechanisms could modulate *fepA* expression in *Y. enterocolitica* and possibly integrate different environmental cues [131]. Although we did not analyze the influence of environmental signals on the OmpR-dependent *fepA* and *fecA* expression, from a physiological point of view, it would be expected that the inhibition of *fepA* and *fecA* by OmpR could alter levels of the encoded receptors in response to environmental cues besides transcriptional control by the Fur repressor. The advantage of limiting FepA and FecA synthesis by OmpR is unclear. Since the solubility of iron increases in the acidic conditions known to activate EnvZ/OmpR in *E. coli* [57,58], the uptake of ferric siderophores by FepA and FecA receptors might be less necessary under these circumstances. In addition, decreasing the level of the outer membrane proteins FepA and FecA, which are known targets of the host immune system [132], may help to limit any host response to *Y. enterocolitica* infection.

With regard to the function of Fur and OmpR in the regulation of *Y. enterocolitica feoA*, we revealed that the former slightly represses *feoA* expression, while the latter weakly activates it. Since our EMSA analysis suggested a poor direct interaction of OmpR with the *feoA* promoter sequence, the positive effect of OmpR on *feoA* expression may result from indirect (by lowering the level of Fur repressor) and weak direct control of *feoA* transcription. Curiously, the positive effect of OmpR on *feoA* expression was also observed in the absence of Fur, and thus it seems that OmpR might also be involved in other regulatory mechanisms not directly connected with the expression of *fur*. Recent studies have shown that a constitutively activated EnvZ/OmpR system caused by a specific mutation in *envZ* (*envZ*_R397L_) induces *feoA/feoB* and downregulates *fepA* and *fecA* expression in *E. coli* [133]. In addition, binding of OmpR to the *feoABC* operon promoter region was indicated. Moreover, these authors hypothesized that the porins OmpC/F and transporter FeoB are involved in the uptake of ferrous ions, leading to an increase in the intracellular Fur-Fe^2+^ level and hence downregulation of *fepA* and *fecA* expression. Thus, although the net regulatory effect of OmpR on *fepA, fecA* and *feoA* expression in *Y. enterocolitica* is consistent with that observed in *E. coli*, the regulatory mechanisms involved might be more complicated. From a physiological point of view, the upregulation of the FeoABC system by OmpR could be important at low pH and in microaerobic or anaerobic environments where ferrous iron dominates.

The results of this study raise questions concerning the adaptive role of OmpR associated with the dual control of different iron transport systems. *Y. enterocolitica* can survive and grow outside and inside the host organism, and this localization influences the nature of the iron available as well as its dedicated transport mechanisms. Thus, the OmpR-mediated induction or repression of the iron uptake system appropriate to the local environment may contribute to the fitness of *Y. enterocolitica.*

Finally, it is noteworthy that studies on pathogenic bacteria have identified Fur as a global regulator that can activate or repress a variety of genes involved in diverse non-iron functions [41,134]. The identification of such direct Fur targets in *Y. enterocolitica* would help to verify the physiological role of the OmpR-mediated inhibition of *fur* expression. Taken together, the presented results lay the foundations for future work to discover further targets for the activity of Fur and thus under OmpR regulatory impact.

## 4. Materials and Methods

### 4.1. Strains, Media and Growth Conditions

The *Y. enterocolitica* and *E. coli* strains and plasmids used in this work are described in Supplementary Table S1. The *Y. enterocolitica* subsp. *palearctica* strain Ye9 of bio-serotype 2/O:9 and its derivatives were used (including its isogenic *ompR* (AR4) and *fur* deletion mutants). *E. coli* strain S17-1 λ*pir* was used as a host for recombinant plasmids. LB broth (Lennox, Sigma Aldrich, St. Louis, MO, USA) and LB0 (10 g/L Tryptone, 5 g/L Yeast Extract) were used as growth media. When necessary, growth media were supplemented with the appropriate antibiotics at the following concentrations: 25 μg/mL chloramphenicol (Cm), 40 µg/mL gentamicin (Gm; 10 µg/mL for *E. coli*), 50 µg/mL kanamycin (Km), 30 µg/mL nalidixic acid (Nal), 12.5 µg/mL tetracycline (Tet). To test the effects of high osmolarity (350 mM NaCl), iron content (10 µM FeCl_3_ or 150 µM 2,2-dipyridyl), oxidative stress (100 µM hydrogen peroxide or 100 µM paraquat) and novobiocin (25 µg/mL), cells were grown overnight (to OD600 ~1.5), diluted to an OD600 of 0.1 and grown for ~4 h to OD600 0.4 when the studied compounds were added. To investigate the effect of acidic conditions, the pH of LB medium was adjusted to 5.6 with MES buffer. Untreated cells were cultured in parallel in an identical fashion to a control.

### 4.2. Molecular Biology Techniques

Standard DNA manipulation methods including polymerase chain reactions (PCRs), restriction digests, ligations and DNA electrophoresis were performed as described previously [135]. Plasmid DNA was isolated using a GeneJET Plasmid Miniprep Kit (Thermo Scientific, Waltham, MA, USA). Genomic DNA was isolated using a Bacterial & Yeast Genomic DNA Purification Kit (EurX, Gdańsk, Poland). PCRs were performed with Phusion High-Fidelity DNA polymerase or DreamTaq DNA polymerase (Thermo Scientific). Oligonucleotide primers used for PCR were purchased from Sigma Aldrich and are listed in Supplementary Table S2. DNA fragments amplified by PCR or obtained by restriction digestion were purified using a PCR/DNA Clean-Up Purification Kit (EurX). DNA sequencing was performed by Genomed S.A. (Warsaw, Poland).

### 4.3. Construction of a Chromosomal Pfur’::lacZYA Fusion

To obtain a P*fur*’::*lacZYA* chromosomal fusion, a 498-bp DNA fragment corresponding to the fur promoter region (−498/−1) was amplified by PCR with the primers LPfurXbaI and PPfurSmaI (Table S2) using *Y. enterocolitica* Ye9N genomic DNA as the template. The obtained fragment was then purified, digested with restriction endonucleases XbaI and SmaI (FastDigest, Thermo Scientific) and ligated into the mobilizable suicide vector pFUSE, cleaved with the same enzymes. The fur promoter fragment was inserted upstream of the *lacZYA* operon in pFUSE to form plasmid pFUSEfur. This construct was introduced into *E. coli* S17-1 λ*pir* by transformation and then transferred into *Y. enterocolitica* strains (Ye9N and the Δ*ompR* mutant AR4) by conjugation. Recombinants harboring plasmid co-integrates were selected on LB agar plus antibiotics (Cm + Nal for strain Ye9N and Cm + Km for strain AR4). The integrity and chromosomal location of the integrated plasmid were confirmed by PCR using primers LFur695 and lacZH991 (Table S2), followed by sequencing of the amplicons.

### 4.4. Construction of Pfur::lacZ Fusions in Plasmid pCM132Gm

Five *fur* promoter fragments of varying length (containing a different number of predicted OmpR-binding motifs) were PCR-amplified using *Y. enterocolitica* Ye9N genomic DNA as template and primers containing unique restriction sites (Table S2). The purified amplicons were digested with restriction endonucleases EcoRI and KpnI and ligated into vector pCM132Gm (derivative of pCM132 [136]) cleaved with the same enzymes, to fuse the promoter fragments to the *lacZ* gene. Plasmids pCMF1234 (−443/+87 relative to the fur translational start site), pCMF234 (−276/+88), pCMF34 (−201/+88), pCMF4 (−173/+88) and pCMF0 (−168/−27) containing promoter fragments of diminishing size and number of putative OmpR-binding sites were constructed using the primer pairs FurAEcoRI/FurBKpnI, FurCEcoRI/FurBKpnI, FurDEcoRI/FurBKpnI, FurEEcoRI/FurBKpnI and FurFEcoRI/FurGKpnI (Table S2), respectively. The obtained promoter-*lacZ* fusions were verified by PCR and DNA sequencing using primers pCM132GmSpr1/pCM132GmSpr2 (Table S2). To obtain an analogous construct carrying the fur promoter region of *E. coli*, a fragment was PCR-amplified with primers FurEcEcoRI/FurEcKpnI using genomic DNA of *E. coli* BW25113 as template. This amplicon and vector pCM132Gm were both digested with EcoRI and KpnI and ligated to produce the construct pCMFEc. The resulting fusion was verified by PCR and sequencing with primers pCM132GmSpr1/pCM132GmSpr2 (Table S2).

### 4.5. Complementation of the ΔompR Mutation

To complement the Δ*ompR* mutation in *Y. enterocolitica* strains, plasmid pBOmpR was used to transform *E. coli* DH5α [74]. Next, pBOmpR was mobilized into recipient *Y. enterocolitica* Δ*ompR* strains by triparental conjugation using *E. coli* DH5α carrying the helper plasmid pRK2013, which provides *tra* and *mob* functions (Table S1). In the case of *E. coli* Δ*ompR* mutant JW3368-1, pBOmpR was introduced by transformation [135].

### 4.6. β-Galactosidase Assay

The modified method of Miller [137] was used to measure β-galactosidase activity in bacterial cells. Overnight cultures of *Y. enterocolitica* or *E. coli*, incubated at 26 or 37 °C, respectively, were diluted to OD600 0.1 in fresh medium and incubation was continued until OD600 0.4 (exponential phase) or overnight (OD600 ~1, early stationary phase). To examine the influence of environmental factors on the activity of the tested promoters, cultures were supplemented with different reagents as described above. After incubation, 80 µL of culture (early stationary phase cultures were diluted to OD600 ~0.4) was mixed with 20 µL of PopCulture Reagent (Merck, Darmstadt, Germany) to disrupt the cell membranes. After 15-min incubation at room temperature, 20 µL of cell lysate was mixed with 130 µL of Z buffer and 30 µL of ONPG (4 mg/mL). The reaction was stopped after sufficient yellow color had developed by adding 75 µL of 1 M Na_2_CO_3_. The absorbance was then measured at both 420 and 550 nm and β-galactosidase activity, expressed in Miller Units, was calculated as described previously [137]. Each assay was performed at least in triplicate.

### 4.7. RT-qPCR

Total RNA was isolated from cultures of *Y. enterocolitica* grown at 26 °C to OD600 ~1 using a NucleoSpin RNA purification kit (Macherey-Nagel, Düren, Germany). The purity and quality of the RNA were assessed using an Agilent RNA 6000 Nano Kit (Agilent Technologies, Santa Clara, CA, USA). The RNAs were DNase-treated using a TURBO DNA-free Kit (Invitrogen, Carlsbad, CA, USA) and 1 µg of each preparation was used for cDNA synthesis with a Maxima H Minus First Strand cDNA Synthesis Kit (Thermo Scientific) using random hexamer primers. The cDNAs were then used as the template for real-time qPCR reactions performed with 5× HOT FIREPol EvaGreen qPCR Mix Plus (Solis Biodyne, Tartu, Estonia) and primers listed in Table S2. Reactions were performed using a LightCycler 480 II (Roche, Basel, Switzerland). Relative quantification of transcripts in various genetic backgrounds was performed using the 2^−^^ΔΔCT^ method with 16S rDNA serving as a reference gene.

### 4.8. Construction of Strains Expressing Fur Carrying a 3×FLAG Epitope

*Y. enterocolitica* strains Ye9Fflag and AR4Fflag, expressing a Fur-3×FLAG fusion protein carrying a 3×FLAG epitope peptide at the C-terminal end, were obtained by homologous recombination using suicide vector pDS132 carrying the sucrose sensitivity system [138]. An in-frame fusion of *fur* with the 3×FLAG epitope coding sequence was constructed by overlap extension PCR using primers listed in Table S2. As the first step, two DNA fragments (A and B) were PCR-amplified using *Y. enterocolitica* Ye9 genomic DNA as the template. Fragment A comprising the 132-bp sequence upstream of *fur* plus the *fur* ORF without a STOP codon (444 bp), with an overhang of 20 bp of the 1×FLAG coding sequence, was amplified using the primer pair 1FurFLAGXba-F/2FurFLAG-R. Fragment B composed of a 3×FLAG coding sequence (69 bp) plus 635 bp downstream of the *fur* STOP codon was amplified with the second pair of primers 3FLAGFur-F/4FLAGFurXba-R. A mixture of these two amplicons was then used as the template in a PCR with the flanking primers 1FurFLAGXba-F and 4FLAGFurXba-R to generate fragment C (1308 bp). This fragment was purified, digested with XbaI and then cloned into the corresponding site of suicide vector pDS132. The resulting construct pDSFur-FLAG was sequenced to confirm the absence of errors and used to transform *E. coli* S17-1 λ*pir*. Plasmid pDSFur-FLAG was then introduced into different *Y. enterocolitica* strains via conjugation. Transconjugants carrying pDSFur-FLAG integrated into the chromosome were selected on LB agar supplemented with Cm (25 µg/mL). To force the second cross-over recombination, strains were plated on LB agar supplemented with 10% sucrose. Sucrose-resistant colonies that had lost chloramphenicol resistance were screened for the 3×FLAG coding sequence using colony PCR with the primer pair FlagSpr1/FlagSpr2. The presence of an intact *fur*::*3×flag* fusion in selected strains Ye9Fflag and AR4Fflag was confirmed by PCR and sequencing using primer pair 0FurFLAG-F/5FurFLAG-R (Table S2).

### 4.9. Western Blotting

For immunological detection of HemR and the FLAG-tagged Fur protein, *Y. enterocolitica* strains were grown in LB medium under the desired conditions until OD600 ~1. Volumes of 1 mL of each culture were then centrifuged and the cell pellets were resuspended in OD600 ×100 μL of Laemmli buffer [135]. Samples were then heated at 95 °C for 10 min to solubilize cellular proteins and separated by electrophoresis on 12% TGX Stain-Free FastCast Acrylamide gels (Bio-Rad, Hercules, CA, USA). Each gel was then Stain-Free-activated by a 10 min exposure to UV and imaged using a GE Healthcare AI600 Imager to confirm equal protein loading in each lane. Next, the proteins were transferred to a nitrocellulose membrane (Amersham Protran 0.2 µm Western Blotting Membrane; GE Healthcare, Chicago, IL, USA) using a wet electroblotting system (100 V for 1 h; Bio-Rad). The membranes were then blocked with 3.5% non-fat dried milk diluted in TBST and treated with rabbit anti-HemR polyclonal antibody (1:10,000; generous gift of Prof. Jürgen Heesemann) [15] or mouse anti-FLAG monoclonal antibody (1:5000; Sigma Aldrich). Sheep anti-rabbit IgG conjugated with horseradish peroxidase (HRP) was used as the secondary antibody (1:15,000; Promega, Madison, WI, USA) to detect anti-HemR antibody, while sheep anti-mouse IgG conjugated with HRP was used as the secondary antibody (1:8000; Sigma Aldrich) with the anti-FLAG antibody. Positive immunoreaction was visualized using Clarity Western ECL Blotting Substrate (Bio-Rad) for HRP-based chemiluminescent detection with a GE Healthcare AI600 Imager. As a further protein loading control, membranes were also probed with an antiserum specific for the cytoplasmic molecular chaperone DnaK (rabbit anti-DnaK, 1:10,000; kind gift of Prof. Sabina Kędzierska-Mieszkowska, University of Gdansk, Poland) [139]. The immunoreactive band intensities were quantified by densitometric analysis using ImageQuant TL analysis software (GE Healthcare).

### 4.10. Electrophoretic Mobility Shift Assays (EMSAs)

Overproduction and purification of recombinant OmpR-His_6_ for in vitro DNA binding studies were performed as described previously [80]. Briefly, *Y. enterocolitica* OmpR protein with a His_6_ motif at its N terminus was expressed from plasmid pETOmpR in *E. coli* BL21(DE3) and purified using Ni-NTA resin (Qiagen, Hilden, Germany). The concentration of the purified OmpR was determined using the RC DC protein assay (Bio-Rad). DNA fragments comprising the regulatory regions upstream of *fur*, *fecA*, *fepA* and *feoA* of *Y. enterocolitica* and *fur* of *E. coli* were PCR-amplified using primers listed in Table S2, with the respective genomic DNAs as template. The amplicons were purified using a Gene Matrix PCR/DNA Clean-Up Kit (EurX) and the concentration of DNA was determined with a NanoDrop 2000 spectrophotometer (Thermo Scientific). EMSA reactions were carried out in 10 μL of OmpR-binding buffer (50 mM Tris-HCl pH 8.0, 100 mM KCl, 1 mM EDTA, 1 mM DTT, 20 mM MgCl_2_, 12% glycerol, 100 µg/mL BSA, 0.1% Triton X-100) and contained appropriate DNA fragments of the studied promoters (0.05 or 0.3 pmol) with increasing amounts of OmpR-His_6_. DNA probes were mixed with OmpR-His_6_ with the molar ratio of 1:20–910. The purified protein was used in EMSAs without in vitro phosphorylation. To confirm binding specificity, a 304-bp or a 211-bp fragment of *Y. enterocolitica* Ye9 16S rDNA generated by PCR was included as a non-specific competitor in some binding reactions (Table S2). OmpR binding to the DNA targets was performed at 22 °C for 15 min, then 2 µL of 30% (*v*/*v*) glycerol was added and the samples were separated on 4.2% native polyacrylamide gels (19:1 acrylamide/bisacrylamide, 0.2×TBE) containing 2% glycerol. To improve the visualization of slower migrating OmpR/DNA complexes, non-denaturing polyacrylamide gels comprised of two layers were used, i.e., a large pore 4.2% gel on the top and a small pore 8.4% gel on the bottom. All gels were pre-run in 0.2×TBE running buffer for 40 min at 80 V. The samples were then loaded, and electrophoresis continued at 110 V for approximately 3 h. The gels were then stained in 1×SYBRgreen solution (Invitrogen) and visualized using a GE Healthcare AI600 imager.

### 4.11. Bioinformatic and Statistical Analyses

In silico analyses of the *fur* locus were performed on a shotgun genome sequence of *Y. enterocolitica* subsp. *palearctica* Ye9N (bio-serotype 2/O:9; NCBI/GenBank: JAALCX000000000). BLAST analysis (https://blast.ncbi.nlm.nih.gov/BlastAlign.cgi) and Clustal Omega (https://www.ebi.ac.uk/Tools/msa/clustalo/) were used for sequence alignment. Promoter prediction was performed using the software BPROM [81]. Statistical analyses were performed using Prism 7 software (v.7.02, GraphPad, San Diego, CA, USA). One-way ANOVA was used to determine statistically significant differences.

## 5. Conclusions

The human enteropathogen *Y. enterocolitica* is characterized by various iron transport systems that are important for the pathogenic abilities of this species. This study revealed the importance of the two-component system response regulator OmpR in the regulation of iron homeostasis in *Y. enterocolitica* by modulation of the level of Fur, the main regulator of iron transport and metabolism. It also established that OmpR can directly influence the expression of ferric and ferrous iron uptake genes and hence the level of iron in *Y. enterocolitica* cells. This dual control via OmpR probably plays a role in the precise expression of the appropriate iron uptake system, according to the ecological niche of the host, and contributes to the fitness and virulence of *Y. enterocolitica*. These findings expand the role of the OmpR regulator in the physiology of *Y. enterocolitica*.

## Data Availability

The data that support the findings of this study are available from the corresponding author upon reasonable request.

## Supplementary Materials

Supplementary Materials can be found at https://www.mdpi.com/1422-0067/22/3/1475/s1.
