# Supplementary material for: The Regulator OmpR in Yersinia enterocolitica Participates in Iron Homeostasis by Modulating Fur Level and Affecting the Expression of Genes Involved in Iron Uptake
Source: Int J Mol Sci. 2021 Feb 2;22(3):1475. doi: 10.3390/ijms22031475 (PMC7867234; doi:10.3390/ijms22031475)
Supplement: Supplementary file 1 [file ijms-22-01475-s001.pdf]

Table S1. Strains and plasmids used in this study.

Strains and plasmids	Description	Reference or source
***Y. enterocolitica* 2/O:9 strains**		
Ye9	clinical isolate of serotype O:9, carrying virulence plasmid pYV	Clinical isolate, laboratory collection
Ye9N	Ye9 derivative, spontaneous Nal^R mutant	[1]
AR4	Ye9N derivative Δ*ompR*::Km defective in OmpR production, Nal^R, Km^R	[2]
Ye9*fur*	Ye9N derivative Δ*fur*::Gm defective in Fur production, Nal^R, Gm^R	[3]
AR4*fur*	Ye9N derivative Δ*fur*::Gm and Δ*ompR*::Km defective in Fur and OmpR production, Nal^R, Gm^R, Km^R	[3]
Ye9F	Ye9N, P*fur*'::*lacZYA*, Nal^R, Cm^R	This study
AR4F	AR4, P*fur*'::*lacZYA*, Nal^R, Km^R, Cm^R	This study
Ye9Fflag	Ye9N, *fur*::*3xflag*	This study
AR4Fflag	AR4, *fur*::*3xflag*	This study
***E. coli* strains**		
DH5α	F', *endA1*, *hsdR17*(r^-_k m^+_k), *supE44*, *thi-1*, *recA1*, Δ(*lacIZYA-argF*) *U169 deoR* [ϕ80dlacΔ(*lacZ*)M15]	[4]
S17-1 λ*pir*	Tp^R, Str^R, *pro*, *thi*, *recA*, *hsdR514*, (r^- m^+), *λpir*, RP4: 2- Tc::Mu-Km^R::Tn7	[5]
BL21 (DE3)	F$^-$, *ompT hsdSB* ($r_{B^-}m_{B^-}$) *gal*, *dcm* (DE3)	Life Technologies
BW25113	F$^-$, *Δ(araD-araB)567*, Δ*lacZ4787*(::rrnB-3), *λ*$^-$, *rph-1*, Δ*(rhaD-rhaB)568*, *hsdR514*	[6]
JW3368-1	F-, *Δ(araD-araB)567*, Δ*lacZ4787(::rrnB-3), λ-*, Δ*ompR739::kan, rph-1,* Δ*(rhaD-rhaB)568, hsdR514*	[6]
**Plasmids**		
pFUSE	suicide vector, derivative of pEP185.2 with promoterless *lacZYA* genes, Cm^R	[7]
pFUSE*fur*	pFUSE with XbaI/SmaI fragment (498 bp) of *fur* promoter region, Cm^R	This study
pBOmpR	pBBR1MCS-3 with XhoI/PstI fragment containing entire coding sequence of *ompR* (ORF with rbs) and 6His-tag region Tet^R, previously named as pBR3	[1]
pBBR1 MCS-3	broad-host-range cloning vector, *ori* pBBR1, Mob, *oriT* RK2, Tet^R	[8]
pRK2013	Helper plasmid, Km^R	[9]
pCM132Gm	pCM132 derivative, *ori* pMB1, *ori* RK2, *oriT* RK2, promoterless *lacZ* gene, Gm^R	DBG collection*
pCMF1234	pCM132Gm derivative carrying 442 bp upstream of *fur* start codon and 88 bp of *fur* ORF cloned upstream of promoterless *lacZ* gene between EcoRI and KpnI sites	This study
pCMF234	pCM132Gm derivative carrying 276 bp upstream of *fur* start codon and 88 bp of *fur* ORF cloned upstream of promoterless *lacZ* gene between EcoRI and KpnI sites	This study
pCMF34	pCM132Gm derivative carrying 201 bp upstream of *fur* start codon and 88 bp of *fur* ORF cloned upstream of promoterless *lacZ* gene between EcoRI and KpnI sites	This study
pCMF4	pCM132Gm derivative carrying 173 bp upstream of *fur* start codon and 88 bp of *fur* ORF cloned upstream of promoterless *lacZ* gene between EcoRI and KpnI sites	This study
pCMF0	pCM132Gm derivative carrying -168/-27 bp fragment upstream of *fur* start codon cloned upstream of promoterless *lacZ* gene between EcoRI and KpnI sites	This study
pCMFEc	pCM132Gm derivative carrying 242 bp upstream of *fur* start codon and 8 bp of *fur* ORF cloned upstream of promoterless	This study

	lacZ gene between EcoRI and KpnI sites	
pDS132	*ori* R6K (narrow host range, replication only in *E. coli* λ*pir*), *oriT* RK2, *sacB*, Cm^R	[10]
pDSFur-FLAG	pDS132 carrying 132 bp upstream of *fur* start codon and 444 bp of *fur* ORF (without stop codon) fused in frame with 69 bp of 3xFLAG epitope and 663 bp downstream of *fur* stop codon	This study
pETOmpR	pET28a carrying the entire *ompR* coding sequence (725 bp fragment), Km^R	[11]
*Department of Bacterial Genetics, Institute of Microbiology, Faculty of Biology, University of Warsaw		
Cm^R chloramphenicol resistance, Gm^R gentamicin resistance, Km^R kanamycin resistance, Nal^R nalidixic acid resistance, Sm^R streptomycin resistance, Tet^R tetracycline resistance, ::Km, insertion of kanamycin resistance cassette; ::Gm, insertion of gentamicin resistance cassette		

Table S2. Oligonucleotide primers used in this study.

Purpose and Target	Name of primer	Primer sequence (5'→ 3')*	Restriction enzyme	Reference
Construction of P*fur*'::*lacZYA* chromosomal, transcriptional fusions	LPfurXbaI	tgtctagaGGCGGATGACGATCACTTTA	XbaI	This study
	PPfurSmaI	tacccgggGCGGATTCAGTCCTGTTACT	SmaI	This study
Confirmation the correct sequence of P*fur*'::*lacZYA* chromosomal fusion	LFur695	GATTTCTTCCCGACACTGGA		This study
	lacZH991	CATCGCAGGCTTCTGCTTC		This study
Construction of YeP*fur*::*lacZ* transcriptional fusions with **four** OmpR-binding sites	FurAEcoRI	tagaattcTGACAAATGAGCGCGTTGAA	EcoRI	This study
Construction of YeP*fur*::*lacZ* transcriptional fusions with **four/three/two/one** OmpR-binding sites	FurBKpnI	taggtaccCCGGATCTTGCAACACTTCC	KpnI	This study
Construction of YeP*fur*::*lacZ* transcriptional fusions with **three** OmpR-binding sites	FurCEcoRI	tagaattcCAGATCCGCACTTTCATCCG	EcoRI	This study
Construction of YeP*fur*::*lacZ* transcriptional fusions with **two** OmpR-binding sites	FurDEcoRI	tagaattcACTTAAATTATGCAACAATCCCATTC	EcoRI	This study
Construction of YeP*fur*::*lacZ* transcriptional fusions with **one** OmpR-binding site	FurEEcoRI	tagaattcCAATACATTGCATGATTAATCGGGC	EcoRI	This study
Construction of YeP*fur*::*lacZ* transcriptional fusions **without** OmpR-binding sites	FurFEcoRI	tagaattcCATTGCATGATTAATCGGGCAAAG	EcoRI	This study
	FurGKpnI	taggtaccCAAATTAAGGCTGTTCAGCCTATG	KpnI	This study
Construction of EcP*fur*::*lacZ* transcriptional fusions (promoter from *E. coli*)	FurEcEcoRI	tggaattcCCAATCAAAATAATTGCTACAAATTT	EcoRI	This study
	FurEcKpnI	tgggtaccTCAGTCATGCGGAATCTGTC	KpnI	This study
Confirmation the correct sequence of fusions constructed in pCM132Gm	pCM132GmSpr1	CTGCAAGGCGATTAAGTTGG		[12]
	pCM132GmSpr2	CATAAACTGCCAGGCATCAA		
Construction of strains carrying Fur-3xFLAG	1FurFLAGXba-F	gctctagaAACAACCACGGTTTTCATTTGGGG	XbaI	This study
	2FurFLAG-R	ccgtcatggtctttgtagtcTCTTTTATCGTGCGCAGATTCATTTTCACG		This study
	3FLAGFur-F	gactacaaagaccatgacggtgattataaagatcatgatatcgattacaagga tgacgatgacaagtagTGTCGTTTTATCGCGTTGACTTAT		This study
	4FLAGFurXba-R	gctctagaAGTTGTTGGCGGGGAATTGG	XbaI	This study
	FlagSpr1	TCATCGTCATCCTTGTAATCG		This study

	FlagSpr2	CTACAAAGACCATGACGGTGA		This study
	0FurFLAG-F	AAATCCGCACTTTCATCCGT		This study
	5FurFLAG-R	GTATTACCACTGGCCGAACG		This study
EMSA, fragment F1234	EFurYe1	TGACAAATGAGCGCGTTGAA		This study
	EFurYe2	CATGCGGATTCAGTCCTGTT		This study
EMSA, fragment F123	EFurYe1	sequence above		This study
	EFurYe3	TTGCGTCTCATTATAGAACTGCT		This study
EMSA, fragment F234	EFurYe4	CGTTGCTTCTATCCCTCTGG		This study
	EFurYe2	sequence above		This study
EMSA, fragment F12	EFurYe1	sequence above		This study
	EFurYe5	ATGACTGCAAAGTATAAGGTTACAAA		This study
EMSA, fragment F23	EFurYe4	sequence above		This study
	EFurYe3	sequence above		This study
EMSA, fragment F34	EFurYe6	GCAGTCATATACTTAAATTATGCAACA		This study
	EFurYe2	sequence above		This study
EMSA, fragment F1	EFurYe7	AGAACTGAATCTGGCCGAAA		This study
	EFurYe8	TCATTTTGCACGGATGAAAG		This study
EMSA, fragment F2	EFurYe4	sequence above		This study
	EFurYe5	sequence above		This study
EMSA, fragment F3	EFurYe6	sequence above		This study
	EFurYe3	sequence above		This study
EMSA, fragment F4	EFurYe9	CAATACATTGCATGATTAATCGGGC		This study
	EFurYe2	sequence above		This study
EMSA, fragment F0	EFurYe10	GCATGATTAATCGGGCAAAG		This study
	EFurYe11	GGCTGTTCAGCCTATGACATC		This study
EMSA, 211-bp fragment of 16S rDNA used as a negative control	E16S211Ye-F	TACGCATTTCACCGCTAC		[3]
	E16S211Ye-R	CAGAAGAAGCACCGGCT		
EMSA, 304-bp fragment of 16S rDNA used as a negative control	E16S304Ye-F	ATTCCGATTAACGCTTGCAC		[11]
	E16S304Ye-R	GTGGGGTAATGGCTCACCTA		
EMSA, fragment F_{Ec} (for *E. coli*)	EFurEcF	TGTGATGCGGCGTAGACTCA		This study
	EFurEcR	GCTTAGTAACAGGACAGATTCCGC		This study
EMSA, promoter *fecA*	EFecAYe-F	CCCCCTTATTCCAAATGGTT		This study
	EFecAYe-R	CAGAGACAAACGGGAAAACG		This study
EMSA, promoter *fepA*	EFepAYe-F	TGCATAATTTGGTCGCGATA		This study
	EFepAYe-R	CAATCTATTCACGGGGCAAC		This study
EMSA, promoter *feoA*	EFeoAYe-F	GGTGGTAAAACAAGCGCAAA		This study
	EFeoAYe-R	ATTTTGTAGGAGCGCTGTGG		This study
RT-qPCR analysis of *Y. enterocolitica fecA*	RT*fecA*Ye9-F	CTTGACGGCTGAAAAAGCACA		This study

expression	RT*fecA*Ye9-R	TGAATGCCAACTCCACACCT		This study
RT-qPCR analysis of *Y. enterocolitica fepA* expression	RT*fepA*Ye9-F	ATGCGGTGCGTTATGGTTG		This study
	RT*fepA*Ye9-R	TGATATTCACCACGCCACCT		This study
RT-qPCR analysis of *Y. enterocolitica feoA* expression	RT*feoA*Ye9-F	TGCTTCCCGGCTCTTCATTT		This study
	RT*feoA*Ye9-R	GTTGCAGGTCTAGCGTCAGT		This study
RT-qPCR analysis of *Y. enterocolitica fur* expression	RT*fur*Ye9-F	CGGTATTGTTACCCGCCATAA		This study
	RT*fur*Ye9-R	TCACTTTGCCGCAATCCA		This study
RT-qPCR analysis of *Y. enterocolitica* gene expression – internal control 16s rRNA	RT16rRNAYe9-F	CATCATGGCCCTTACGAGTAG		This study
	RT16rRNAYe9-R	CCGGACTACGACAGACTTTATG		This study

*- 5’ extensions added to the primers are shown as lowercase

REFERENCES

1. Brzostek, K.; Brzostkowska, M.; Bukowska, I.; Karwicka, E.; Raczkowska, A. OmpR negatively regulates expression of invasin in *Yersinia enterocolitica*. *Microbiology* **2007**, *153*, 2416–2425, doi:10.1099/mic.0.2006/003202-0.
2. Brzostek, K.; Raczkowska, A.; Zasada, A. The osmotic regulator OmpR is involved in the response of *Yersinia enterocolitica* O:9 to environmental stresses and survival within macrophages. *FEMS Microbiol. Lett.* **2003**, *228*, 265–271, doi:10.1016/S0378-1097(03)00779-1.
3. Jaworska, K.; Nieckarz, M.; Ludwiczak, M.; Raczkowska, A.; Brzostek, K. OmpR-Mediated Transcriptional Regulation and Function of Two Heme Receptor Proteins of *Yersinia enterocolitica* Bio-Serotype 2/O:9. *Front. Cell. Infect. Microbiol.* **2018**, *8*, 333, doi:10.3389/fcimb.2018.00333.
4. Sambrook, J.; Russel, D. *Molecular Cloning: A Laboratory Manual*; 3rd ed.; Cold Spring Harbor Laboratory Press.: Cold Spring Harbor, NY, 2001;
5. Simon, R.; Priefer, U.; Pühler, A. A Broad Host Range Mobilization System for In Vivo Genetic Engineering: Transposon Mutagenesis in Gram Negative Bacteria. *Bio/Technology* **1983**, *1*, 784–791, doi:10.1038/nbt1183-784.
6. Baba, T.; Ara, T.; Hasegawa, M.; Takai, Y.; Okumura, Y.; Baba, M.; Datsenko, K.A.; Tomita, M.; Wanner, B.L.; Mori, H. Construction of *Escherichia coli* K-12 in-frame, single-gene knockout mutants: the Keio collection. *Mol. Syst. Biol.* **2006**, *2*, 2006.0008, doi:10.1038/msb4100050.
7. Baumler, A.J.; Tsolis, R.M.; van der Velden, A.W.; Stojiljkovic, I.; Anic, S.; Heffron, F. Identification of a new iron regulated locus of *Salmonella typhi*. *Gene* **1996**, *183*, 207–213, doi:10.1016/s0378-1119(96)00560-4.
8. Kovach, M.E.; Elzer, P.H.; Hill, D.S.; Robertson, G.T.; Farris, M.A.; Roop, R.M. 2nd; Peterson, K.M. Four new derivatives of the broad-host-range cloning vector pBBR1MCS, carrying different antibiotic-resistance cassettes. *Gene* **1995**, *166*, 175–176, doi:10.1016/0378-1119(95)00584-1.
9. Figurski, D.H.; Helinski, D.R. Replication of an origin-containing derivative of plasmid RK2 dependent on a plasmid function provided in trans. *Proc. Natl. Acad. Sci. U. S. A.* **1979**, *76*, 1648–1652, doi:10.1073/pnas.76.4.1648.
10. Philippe, N.; Alcaraz, J.-P.; Coursange, E.; Geiselmann, J.; Schneider, D. Improvement of pCVD442, a suicide plasmid for gene allele exchange in bacteria. *Plasmid* **2004**, *51*, 246–255, doi:10.1016/j.plasmid.2004.02.003.
11. Nieckarz, M.; Raczkowska, A.; Debski, J.; Kistowski, M.; Dadlez, M.; Heesemann, J.; Rossier, O.; Brzostek, K. Impact of OmpR on the membrane proteome of *Yersinia enterocolitica* in different environments: repression of major adhesin YadA and heme receptor HemR. *Environ. Microbiol.* **2016**, *18*, 997–1021, doi:10.1111/1462-2920.13165.
12. Nieckarz, M.; Raczkowska, A.; Jaworska, K.; Stefańska, E.; Skorek, K.; Stosio, D.; Brzostek, K. The Role of OmpR in the Expression of Genes of the KdgR Regulon Involved in the Uptake and Depolymerization of Oligogalacturonides in *Yersinia enterocolitica*. *Front. Cell. Infect. Microbiol.* **2017**, *7*, 366, doi:10.3389/fcimb.2017.00366.

(A)

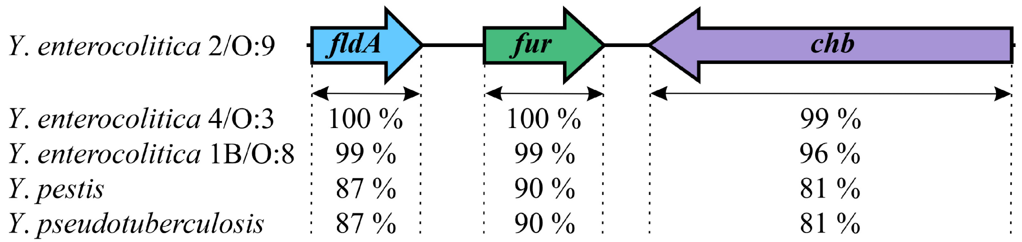

(B)

```
Y. pseudotuberculosis        ATCTCCCCGCAACAA-GAAACGCTCTGCGATAGTATTCACAGCAGAGCGTTTCTGTTCCA   59
Y. pestis                    ATCTCCCCGCAACAA-GAAACGCTCTGCGATAGTATTCACAGCAGAGCGTTTCTGTTCCA   59
Y. enterocolitica 1B/O:8     TTGTCATGTCGGAAACGCGGGGATGAAATTTAATATTG-CCACGCGTTTCCGTTGCTTCT   59
Y. enterocolitica 2/O:9      TTGTCATGTCGGAAACGCGAGGATGAAATTTAATATTG-CCACGCGTTTCCGTTGCTTCT   59
Y. enterocolitica 4/O:3      TTGTCATGTCGGAAACGCGAGGATGAAATTTAATATTG-CCACGCGTTTCCGTTGCTTCT   59
                              * **    *   ** *    * *      ** ****  *  *          ** * *
                                                          M1

Y. pseudotuberculosis        GTCACTGGCACCCCGCTTTGTTAAGCCGCCCACAGATCCGCATATTCGTCTGGGCAAAAT   119
Y. pestis                    GTCACTGGCACCCCGCTTTGTTAAGCCGCCCACAGATCCGCATATTCGTCTGGGCAAAAT   119
Y. enterocolitica 1B/O:8     ATCCCTCTGGTTATACTCTGTTAAGCCACCCACAAATCCGCACTTTCATCCGTGCAAAAT   119
Y. enterocolitica 2/O:9      ATCCCTCTGGTTATACTCTGTTAAGCCACCCACAGATCCGCACTTTCATCCGTGCAAAAT   119
Y. enterocolitica 4/O:3      ATCCCTCTGGTTATACTCTGTTAAGCCACCCACAGATCCGCACTTTCATCCGTGCAAAAT   119
                              ** **         ** ********* ****** *******  *** ** * *******

Y. pseudotuberculosis        GAAAATAAACCGCTACAAGTTTGTAACTTGACATCTGAGCCCTGTACACTCAAGTGATGT   179
Y. pestis                    GAAAATAAACCGCTACAAGTTTGTAACTTGACATCTGAGCCCTGTACACTCAAGTGATGT   179
Y. enterocolitica 1B/O:8     GAAAATAAACTGCTACAAATTTGTAACCTTATACTTTGCAGTCATATACTTAAATTATGC   179
Y. enterocolitica 2/O:9      GAAAATAAACTGCTACAAATTTGTAACCTTATACTTTGCAGTCATATACTTAAATTATGC   179
Y. enterocolitica 4/O:3      GAAAATAAATTGCTACAAATTTGTAACCTTATACTTTGCAGTCATATACTTAAATTATGC   179
                             *********  ******* ******** * * *  *        ** *** ** * ***
                                           M2                                       M3

Y. pseudotuberculosis        GTTTCCCCCATCCATCAATACACTGCATGATTAATCTTACAAAGTGTGCAATGATATCTT   239
Y. pestis                    GTTTCCCCCATCCATCAATACACTGCATGATTAATCTTACAAAGTGTGCAATGATATCTT   239
Y. enterocolitica 1B/O:8     AACAATCCCATTCATCAATACATTGCATGATTAATCGGGCAAAGTGTGCAATTATATCTT   239
Y. enterocolitica 2/O:9      AACAATCCCATTCATCAATACATTGCATGATTAATCGGGCAAAGTGTGCAATTATATCTT   239
Y. enterocolitica 4/O:3      AACAATCCCATTCATCAATACATTGCATGATTAATCGGGCAAAGTGTGCAATTATATCTT   239
                                   ***** ********** *************   ************* *******

Y. pseudotuberculosis        TGTTAACAACCACCGTTTTCATTTGGGGCTAGCAGCTCTATAATAAGACGCAATTATGAT   299
Y. pestis                    TGTTAACAACCACCGTTTTCATTTGGGGCTAGCAGCTCTATAATAAGACGCAATTATGAT   299
Y. enterocolitica 1B/O:8     TGTTAACAACCACGGTTTTCATTTGGGGCTAGCAGTTCTATAATGAGACGCAATTGAGAA   299
Y. enterocolitica 2/O:9      TGTTAACAACCACGGTTTTCATTTGGGGCTAGCAGTTCTATAATGAGACGCAATTGAGAA   299
Y. enterocolitica 4/O:3      TGTTAACAACCACGGTTTTCATTTGGGGCTAGCAGTTCTATAATGAGACGCAATTGAGAA   299
                             ************* ********************* ******** **********  **
                                                                      Fur box

Y. pseudotuberculosis        TATTGCGCCACGGATGTCATAGGCTAAACAGCCTTAATTTGAATCGATTGTAACAGGACT   359
Y. pestis                    TATTGCGCCACGGATGTCATAGGCTAAACAGCCTTAATTTGAATCGATTGTAACAGGACT   359
Y. enterocolitica 1B/O:8     TATTGCGCCACGGATGTCATAGGCTGAACAGCCTTAATTTGAATCGATAGTAACAGGACT   359
Y. enterocolitica 2/O:9      TATTGCGCCACGGATGTCATAGGCTGAACAGCCTTAATTTGAATCGATAGTAACAGGACT   359
Y. enterocolitica 4/O:3      TATTGCGCCACGGATGTCATAGGCTGAACAGCCTTAATTTGAATCGATAGTAACAGGACT   359
                             ************************* ********************** ***********
                                                                           M4

Y. pseudotuberculosis        GAATCCGCATG       370
Y. pestis                    GAATCCGCATG       370
Y. enterocolitica 1B/O:8     GAATCCGCATG       370
Y. enterocolitica 2/O:9      GAATCCGCATG       370
Y. enterocolitica 4/O:3      GAATCCGCATG       370
                             ***********
```

Supplementary Figure S1. Comparison of the genomic organization of the *fur* locus and the *fur* promoter region of *Yersinia* strains (A) Comparison of the genomic organization of the *fur* locus of *Y. enterocolitica* subsp. *palearctica* Ye9N (2/O:9, NCBI/GenBank Acc. No. NZ_JAALCX000000000.1) with the equivalent loci of *Y. enterocolitica* subsp. *palearctica* Y11 (4/O:3, NCBI/GenBank Acc. No. FR729477.2), *Y. enterocolitica* subsp. *enterocolitica* 8081 (1B/O:8, NCBI/GenBank Acc. No. AM286415.1), *Y. pestis* KIM 10+ (NCBI/GenBank Acc. No. NC_004088.1) and *Y. pseudotuberculosis* NCTC10275 (NCBI/GenBank Acc. No. NZ_LR134373.1). The directions of transcription are indicated by arrows. The encoded products are *fur* – Fur (ferric uptake regulator); *fldA* – flavodoxin I; *chb* – chitobiase. The percentage identities of the genes comprising the *fur* locus of *Y. enterocolitica* Ye9N with homologous sequences of other *Yersinia* strains are shown. (B) *In silico* analysis of *fur* promoter regions in *Yersinia* strains. Similar nucleotides are marked by asterisks. Potential OmpR and Fur binding sites are boxed blue and green, respectively. The first codon of the *fur* gene is marked by a pink box. The nucleotide BLAST (BlastN) program, available on the NCBI website (blast.ncbi.nlm.nih.gov/Blast.cgi), was used for sequence alignment. Clustal Omega (https://www.ebi.ac.uk/Tools/msa/clustalo/) was used for multiple sequence alignment.

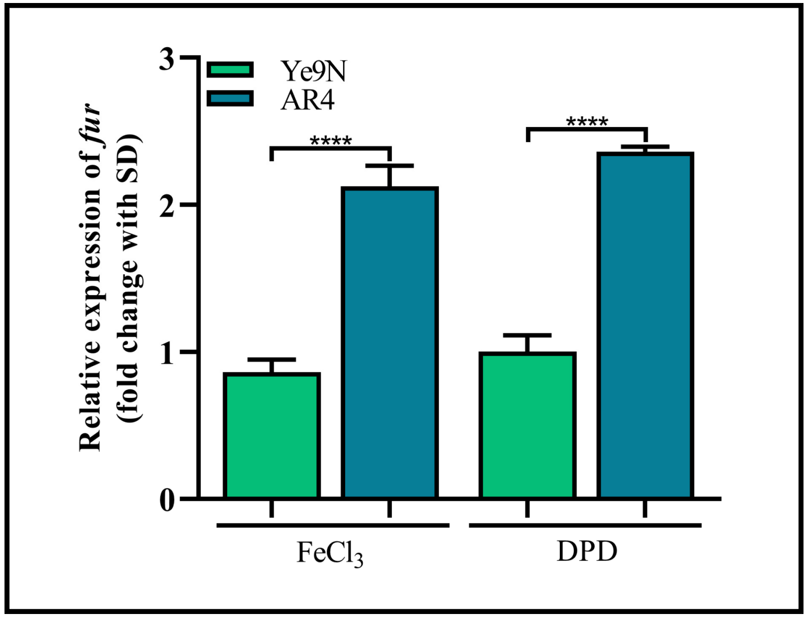

Supplementary Figure S2. OmpR-dependent regulation of *fur* transcription in *Y. enterocolitica* Ye9N**.** Relative *fur* mRNA levels determined by RT-qPCR. This analysis was performed using RNA prepared from cells of the wild-type strain Ye9N and the Δ*ompR* mutant AR4 grown to early stationary phase in LB medium supplemented with 10 µM $FeCl_3$ or 150 µM DPD. Relative *fur* transcript levels, normalized to the amount of 16S rRNA are shown, taking the mRNA level in Ye9N/DPD as 1. The mean value and SD obtained from at least three independent experiments are indicated for each strain.

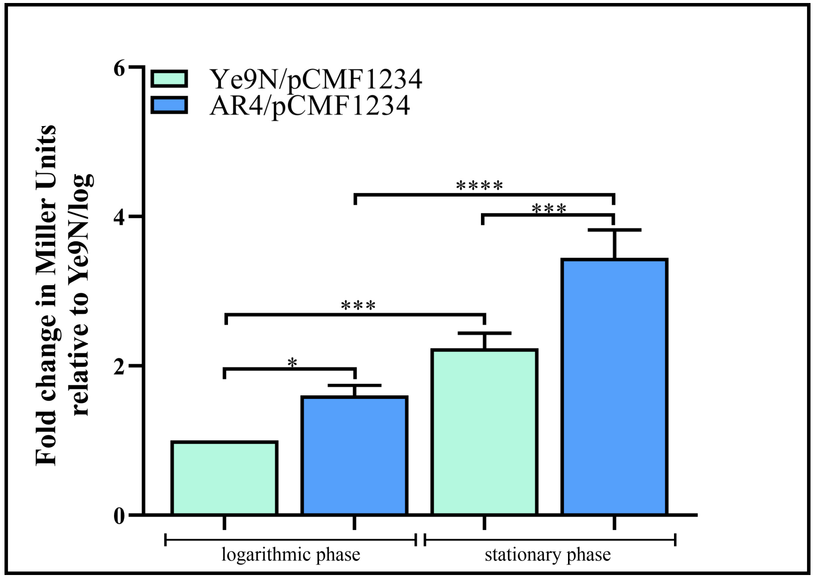

Supplementary Figure S3. Effect of OmpR and growth phase on *fur* expression in *Y. enterocolitica* strains. Expression of plasmid-encoded YeP*fur*::*lacZ* transcriptional fusion in strains Ye9N (WT) and AR4 (Δ*ompR*) is shown. β-Galactosidase activity was measured in strains grown in LB medium supplemented with 10 µM $FeCl_3$. The presented data represent mean β-galactosidase activity values with SD from three independent experiments, each performed using at least triplicate cultures of each strain. Significance was calculated using one-way ANOVA [* $P<0.05$, *** $P < 0.001$, ****$P < 0.0001$].
